# Activated TLR Signaling in Atherosclerosis among Women with Lower Framingham Risk Score: The Multi-Ethnic Study of Atherosclerosis

**DOI:** 10.1371/journal.pone.0021067

**Published:** 2011-06-16

**Authors:** Chiang-Ching Huang, Kiang Liu, Richard M. Pope, Pan Du, Simon Lin, Nalini M. Rajamannan, Qi-Quan Huang, Nadereh Jafari, Gregory L. Burke, Wendy Post, Karol E.Watson, Craig Johnson, Martha L. Daviglus, Donald M. Lloyd-Jones

**Affiliations:** 1 Department of Preventive Medicine, Feinberg School of Medicine, Northwestern University, Chicago, Illinois, United States of America; 2 Rheumatology Division, Feinberg School of Medicine, Northwestern University, Chicago, Illinois, United States of America; 3 Bioinformatics Core, Feinberg School of Medicine, Northwestern University, Chicago, Illinois, United States of America; 4 Cardiology Division, Department of Medicine, Feinberg School of Medicine, Northwestern University, Chicago, Illinois, United States of America; 5 Genomics Core, Feinberg School of Medicine, Northwestern University, Chicago, Illinois, United States of America; 6 Division of Public Health Sciences, Wake Forest University Health Sciences, Winston-Salem, North Carolina, United States of America; 7 Johns Hopkins University, Baltimore, Maryland, United States of America; 8 Division of Cardiology, UCLA School of Medicine, Los Angeles, California, United States of America; 9 Collaborative Health Studies Coordinating Center, University of Washington, Seattle, Washington, United States of America; South Texas Veterans Health Care System, United States of America

## Abstract

**Background:**

Atherosclerosis is the leading cause of cardiovascular disease (CVD). Traditional risk factors can be used to identify individuals at high risk for developing CVD and are generally associated with the extent of atherosclerosis; however, substantial numbers of individuals at low or intermediate risk still develop atherosclerosis.

**Results:**

A case-control study was performed using microarray gene expression profiling of peripheral blood from 119 healthy women in the Multi-Ethnic Study of Atherosclerosis cohort aged 50 or above. All participants had low (<10%) to intermediate (10% to 20%) predicted Framingham risk; cases (N = 48) had coronary artery calcium (CAC) score >100 and carotid intima-media thickness (IMT) >1.0 mm, whereas controls (N = 71) had CAC<10 and IMT <0.65 mm. We identified two major expression profiles significantly associated with significant atherosclerosis (odds ratio 4.85; P<0.001); among those with Framingham risk score <10%, the odds ratio was 5.30 (P<0.001). Ontology analysis of the gene signature reveals activation of a major innate immune pathway, toll-like receptors and IL-1R signaling, in individuals with significant atherosclerosis.

**Conclusion:**

Gene expression profiles of peripheral blood may be a useful tool to identify individuals with significant burden of atherosclerosis, even among those with low predicted risk by clinical factors. Furthermore, our data suggest an intimate connection between atherosclerosis and the innate immune system and inflammation via TLR signaling in lower risk individuals.

## Introduction

Cardiovascular disease (CVD) is the leading cause of morbidity and mortality in the United States and the developed world, and it will soon be the leading cause in the developing world. Atherosclerosis accounts for the vast majority of fatal and non-fatal CVD events. Multivariable (MV) risk equations [1], such as the Framingham risk score (FRS), that include traditional CVD risk factors are generally correlated with presence and extent of subclinical atherosclerosis; however, substantial numbers of individuals with low (<10%) to intermediate (10% to 20%) FRS still develop atherosclerosis [2], [3], and the majority of CVD events occur in those with <20% 10-year predicted risk [4]. Refining risk assessment by identifying those apparent outliers who have significant atherosclerotic burden despite low or intermediate predicted risk, and are therefore at higher risk for events than average [5], could help identify novel mechanisms of atherosclerosis development and progression and target preventive therapies to those who are most likely to benefit.

Atherosclerosis is a disease of inflammation characterized by interactions among platelets, leukocytes, and endothelial cells [6], [7], [8]. Dysfunctional endothelium associated with the presence of atheroma expresses surface molecules such as chemokines, chemoattractants and adhesion molecules that are associated with alterations of expression of cell-surface and secretory proteins in circulating leukocytes and platelets. Therefore, peripheral blood can be useful to measure the burden and further understand the molecular mechanisms of atherosclerosis.

In the present study, we performed microarray gene expression profiling of whole blood from a population of healthy women with low to intermediate FRS to test whether gene expression profiles could distinguish those with and without significant atherosclerosis. We further sought to identify genes and pathways associated with significant burden of atherosclerosis among these women predicted to be at lower risk for CVD events.

## Results

### Characteristics of participants

Among all 119 MESA women, 48 had significant subclinical atherosclerosis (cases) and 71 had no evident subclinical atherosclerosis (controls); among those with FRS<10%, there were 39 cases and 69 controls. The characteristics of all 119 participants at the fourth examination with and without evident subclinical atherosclerosis are shown in Table 1. As expected, a greater burden of CVD risk factors was observed among participants with significant subclinical atherosclerosis. Age, smoking, systolic blood pressure, and the use of cholesterol lowering medications were significantly different between the two groups (p<0.05).

**Table 1:** Characteristics of 119 MESA non-diabetic women with low or intermediate Framingham risk scores.

Variable	WithAtherosclerosis(n = 48)	Without Atherosclerosis(n = 71)	P Value*
Framingham Risk Score (%)	5.8±4.5	2.6±2.9	<0.001
Age, years	69.4±6.8	64.3±7.3	<0.001
Race† (%)	54/27/13/6	58/24/14/4	0.93
Body-Mass Index (Kg/m^2^)	28.2±6.0	27.2±5.8	0.38
Systolic BP (mm Hg)	130.6±21.0	116.3±18.4	<0.001
Diastolic BP (mm Hg)	68.7±9.0	66.9±10.5	0.30
Total cholesterol (mg/dL)	200.2±34.3	193.4±31.5	0.28
LDL cholesterol (mg/dL)	117.6±35.5	108.3±28.5	0.14
HDL cholesterol (mg/dL)	60.0±13.1	72.9±16.0	0.28
Triglycerides (mg/dL)	113.1±69.8	112.1±73.4	0.72
hs-CRP‡ (mg/L)	5.6±9.4	2.3±3.2	0.02
Current smoker (%)	50.0	26.7	0.02
Lipid-lowering medication use (%)	47.9	14.1	<0.001
Anti-hypertension medication use (%)	47.9	32.4	0.13

*p-value by t-test or chi-square test as appropriate.

† non-Hispanic white /African American/Hispanic/Chinese.

‡ hs-CRP levels were measured at MESA baseline examination during 2000∼2002.

### Overview of the gene expression profiles

**Figure 1:**
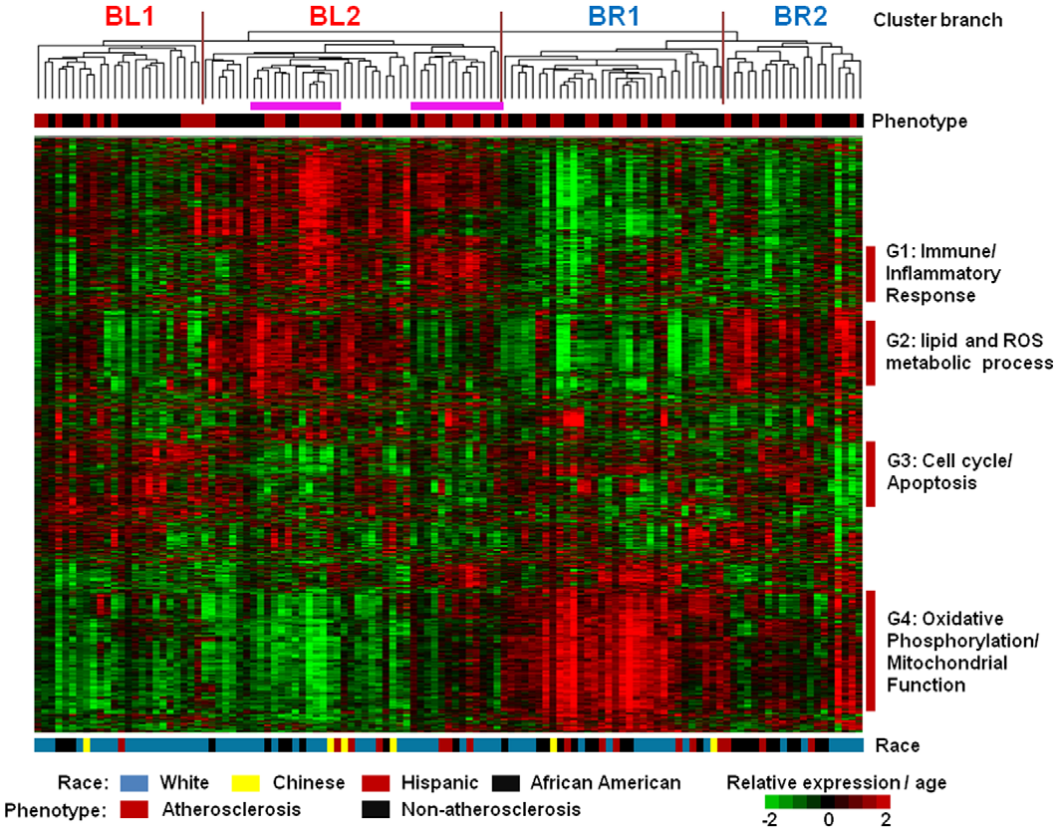
Gene expression patterns among individuals with and without atherosclerosis. 2,057 probes were selected for which expression coefficient of variation was >0.3 and for which at least a half of the 119 microarray samples were detected for the expression values. The unsupervised hierarchical clustering dendrogram shows the relationship among the samples and corresponding gene clusters. Samples have been color-coded by their phenotype (atherosclerosis in red, non-atherosclerosis in black), race (white in blue, Chinese in yellow, Hispanic in red, and African American in black), and age. Age and each probe have been centered on their mean expression values across all samples analyzed. Measurements that are above the mean are colored red and those below the mean are colored green. Groups of genes on the right hand side indicated with colored bars are shown in greater detail and labeled G1–G4. The sample dendrogram shows 4 branches (BL1, BL2, BR1, and BR2). Two subsets (purple bar) under the branch BL2 are enriched with atherosclerosis (both 9/13).

The gene expression patterns differ greatly between cases and controls. The sample dendrogram generated by hierarchical clustering shows that the samples separate into two major branches indicated by BL and BR (Figure 1). The largest cluster of genes (G4) was responsible for separating the samples into these two groups. The G4 cluster contains a large number of genes encoding ribosomal proteins (RPS, RPL, MRPS, MRPL genes) and genes related to oxidative phosphorylation (ATP5J, NDUFA1, A6, A9, B8, S4, COX7C, 7A2, 17), suggesting a role for mitochondrial function and energy metabolism. More African Americans and fewer whites were found in the BR branch than in the BL branch (BR: 38% vs BL: 15% for African American, BR: 40% vs BL: 68% for whites, P = 0.002). More cases were found in the BL branch, however, the association of these two branches with atherosclerosis status did not reach statistical significance (BL vs BR branch: 46% vs 33%, P = 0.19). Each of these two branches further clustered into two sub-branches (BL1, BL2, BR1, and BR2). Two major gene clusters (G1 and G3) distinguish between BL1 and BL2. The majority of genes in G1 are related to immune and inflammatory response (TLR1,2,4,5,6,8, IL1β, IL1R2, IL1RN, NAMPT, FCGR2A, PTGS2) while cell cycle and apoptosis-related genes are found in G3 (BCL2, BAG3, BLK, ATM, MYC, CD27, TNFRSF25). An inverse correlation of gene expression between these two gene clusters is evident. In contrast, the distinct feature between BR1 and BR2 was indicated by gene cluster G2. Several genes involved in lipid and reactive oxygen species metabolic process were discovered (PRDX2, GPX1, GPX4, GLRX5, HAGA, ADIPOR1, OSBP2, CHPT1, PNPLA2). An examination of the prevalence of atherosclerosis among these four sub-branches shows the highest rate in BL2 (22/43, 51%) and lowest rate in BR2 (5/20, 25%), while both the other two branches, BR1 and BL1, had the same rate of 37.5% (9/24 and 12/32). Of note, two subsets indicated by purple bars within the BL2 had the highest rate (both 9/13, 69%) of atherosclerosis. Both subsets are characterized by higher expression of G1 and lower expression of G3, while the subset on the right also had lower expression of G2.

### Association of gene expression profiles with atherosclerosis

The distribution of the 2,057 P-values (Figure S1) using t tests comparing all 48 cases and 71 controls showed a higher frequency of probes at low P values than would be expected by chance alone (229 probes, or 11%, with P<0.05). One hundred and eighty genes were found to have an FDR<24% (215 genes with FDR<20% by SAM) and 20% of genes were estimated to be differentially expressed. This result suggests that a gene signature associated with atherosclerosis may exist; however, the moderate FDR of differentially expressed genes implicates that heterogeneous expression profiles may be present within the case and/or control groups.

The multiple random validation procedure using top 50–60 genes with smallest p-values identified two major gene expression profiling (or molecular) subtypes with an odds ratio of 4.85 (sensitivity 60%, specificity 76%) for the association with atherosclerosis (see Figure S2 and S3). We termed these molecular profiles as Associated with Atherosclerosis (AWA) or not AWA. The odds ratio remained high at 5.3 (sensitivity 62%, specificity 77%) when only the 108 women with FRS <10% were considered. As expected, there was a significantly greater number of differentially expressed genes (1,254, 61%) between these two molecular profiles. As shown in Table 2, age and systolic blood pressure were two risk factors significantly different between those with the two molecular profiles, but less of a difference compared with Table 1. Using multiple logistic regression with adjustment for FRS, the gene expression profiling subtypes remained significantly associated with atherosclerosis with an odds ratio of 3.8 (95% CI  =  [1.6, 8.9], P = 0.002) for all 119 participants and 3.7 (95% CI  =  [1.5, 9.3], P = 0.004) for the 108 low-FRS participants.

**Table 2:** Characteristics of 119 MESA non-diabetic women between two major molecular profiles (e.g., Associate with Atherosclerosis (AWA) or not AWA).

Variable	With AWA profile(n = 46)	Without AWA profile(n = 73)	P Value*
Framingham Risk Score (%)	5.0±4.0	3.1±3.8	0.01
Age, years	68.8±6.5	64.8±7.7	0.003
Race† (%)	59/20/17/4	55/29/11/5	0.58
Body-Mass Index (Kg/m^2^)	27.3±5.8	27.8±5.9	0.62
Systolic BP (mm Hg)	128.2±21.2	118.1±19.4	0.01
Diastolic BP (mm Hg)	69.0±10.3	66.6±9.6	0.21
Total cholesterol (mg/dL)	196.4±32.4	195.9±33.1	0.94
LDL cholesterol (mg/dL)	111.8±33.4	112.2±30.9	0.95
HDL cholesterol (mg/dL)	63.4±14.1	60.6±15.4	0.31
Triglycerides (mg/dL)	105.4±62.6	116.9±77.0	0.38
hs-CRP‡ (mg/L)	5.3±10.1	2.7±2.4	0.08
Current smoker (%)	45.6	30.1	0.13
Lipid-lowering medication use (%)	36.9	21.9	0.12
Anti-hypertension medication use (%)	47.8	31.5	0.11

*p-value by t-test or chi-square test as appropriate.

† non-Hispanic white /African American/Hispanic/Chinese.

‡ hs-CRP levels were measured at MESA baseline examination during 2000∼2002.

A sensitivity analysis was further performed by excluding those on lipid lowering medication, leaving 25 cases and 61 controls for comparison. The multiple random validation procedure on this subset of samples resulted in an association similar to the results from the entire 119 samples (data not shown). Similarly, the association did not change substantially by excluding smokers or those taking medications for hypertension.

### Ontology analysis of differentially expressed genes

**Figure 2:**
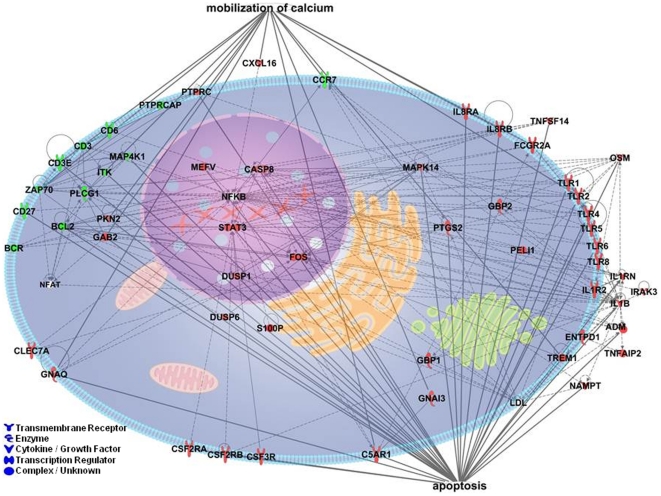
Network representation of the atherosclerosis-associated immune and inflammatory response of peripheral blood. The network consists of 55 genes showing perturbed expression (green, decreased; red, increased expression). This highly connected network is characterized by activation of toll-like receptor and IL-1 signaling (on the right side).

In order to better characterize pathways associated with the AWA profile, we performed ontology analysis on the top 344 probes representing 325 unique genes that had an FDR <0.1% from the comparison between the two molecular profiles. Information on these 325 genes is provided in Table S1. The genes that were identified as associated with the AWA profile strongly represented by members of the innate immune signaling pathway. Genes representing several canonical pathways including Toll-like receptors (TLRs), NF-κB, p38 MAPK, and IL-10 signaling were identified (Table 3). All of these pathways have extensive cross talk in inflammatory signaling. The key genes involved in these pathways include six TLRs (TLR1,2,4,5,6,8), IL1β, IL1RN, IL1R2, IRAK3, and MAPK14. A representation of this immune and inflammatory network from a subset of the 325 differentially expressed genes is shown in Figure 2, in which several genes are also involved in apoptosis and mobilization of calcium. This highly connected network is characterized by activation of the innate immune system, including the TLR and IL-1 signaling pathways (on the right side) and downstream signaling pathways (PTGS2, MAPK14, DUSP1 and DUSP6). Also upregulated are genes that promote myeloid cell growth and survival (CSF2RA, CSF2RB, CSF3R, C5aR1, GBP1, in addition to the TLR and IL-1 signaling molecules). These data suggest that the AWA profile was an Activated Innate Immune Gene (AIIG) signature. The network also shows decreased expression in genes related to adaptive immunity, including the genes important in T cell activation and maturation (CD3, CD3 epsilon, CD27, CD6, and ZAP70) and CCR7 which is important in the recruitment of T cells and myeloid dendritic cells. Consistent with the result from IPA, DAVID bioinformatics analysis also showed enrichment in response to wounding, immune/inflammatory response, leukocyte activation, and TLR signaling. Other than immune response and cytokine and chemokine mediated signaling pathways, PANTHER analysis also revealed 8 genes involved in the apoptosis pathway. Information regarding the enriched functional categories and genes from these bioinformatics analyses are provided in Table S2.

**Table 3:** Atherosclerosis-associated genes and the corresponding enriched canonical pathways identified by Ingenuity Pathway Analysis.

Signaling Pathways	Molecule
Toll-like Receptor	TLR1, TLR2, TLR4, TLR5, TLR6, TLR8, FOS, IRAK3, MAPK14
P38 MAPK	MAPK4K1, MAPK14, IL1β, IL1RN, IL1R2, IRAK3, DUSP1, CREB5
IL-10	FCGR2A, FOS, IL1β, IL1RN, IL1R2, MAPK14, STAT3
NF-κB	IL1β, IL1RN, IL1R2, IRAK3, TGFA, ZAP70, TLR1, TLR2, TLR4, TLR5,
	TLR6, TLR8

### Comparison with gene expression pattern of peripheral blood leukocytes after endotoxin injection

**Figure 3:**
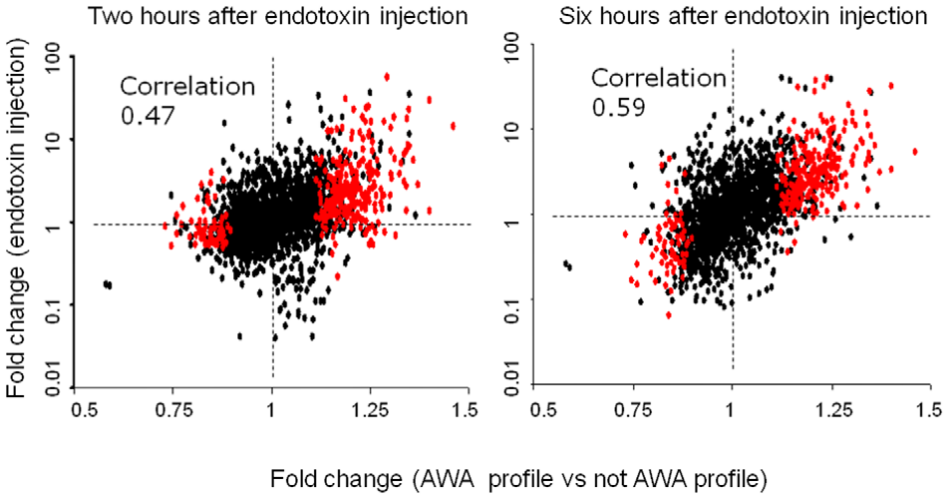
Expression changes of 2,057 probes between acute and chronic inflammation. X-axis: fold changes of gene expression comparing AWA profile with not AWA profile. Y-axis: fold changes of gene expression comparing expression levels before and after endotoxin (LPS) injection. A stronger correlation of fold changes between chronic inflammation and acute inflammation six hours after endotoxin injection was observed. Red dots: 325 genes in the activated innate immune gene signature. AWA: associated with atherosclerosis.

To further validate the observation that TLR pathway is activated in AWA profile, we compared our gene expression data with Calvano’s study[9], which examined the gene expression profiles in blood leukocytes of human subjects who received bacteria endotoxin injection – an established model of acute inflammatory response via TLR activation. For Calvano’s study, fold changes and P values for genes were calculated by comparing expression levels at two or six hours after endotoxin injection with baseline levels. A total of 501 and 583 probes, respectively, were found to have P values <0.05 in both our data and Calvano’s data at two and six hours after endotoxin injection (P<10^−5^ and P<10^−15^). More importantly, a significant concordance of fold changes was observed between both studies (Figure 3), with a stronger correlation of fold change between the two studies at six hours after endotoxin injection than at two hours (Pearson correlation 0.59 vs 0.47, P<10^−15^).

### Confirmation of gene expression data

Quantitative real-time RT-PCR was performed to confirm the RNA expression levels of eight selected genes involved in various functions including lipid metabolism, immune and inflammatory response, apoptosis, cell proliferation, and hypoxia signaling (TNFSF14, TLR8, TLR4, CREB5, IL1β, IL1RN, EGLN1, TGFA). All eight genes showed high correlation (0.47∼0.65, P = 5.6•10^−8^∼1.8•10^−15^, see Table S3) between the microarray expression and RT-PCR measures.

### The association of AIIG signature with atherosclerosis among men

**Figure 4:**
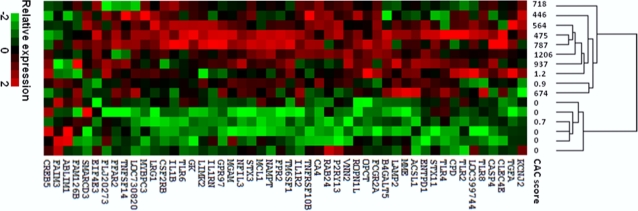
Association of expression pattern with coronary artery calcium (CAC) among men. Cluster analysis of 16 selected men from the CHAS cohort was performed using top 50 genes from the activated innate immune gene (AIIG) signature.

We further characterized the association of atherosclerosis with the AIIG signature described above, employing peripheral blood from 16 white men aged 65–80. The clinical characteristics of these 16 men are shown in Table S4. Most of CVD risk factors are comparable between the two CAC groups. However, lipid-lowering medication use is significantly higher among those with high CAC (75% vs 25%, P = 0.05) while their total cholesterol levels were lower (154 mg/dL vs 193 mg/dL, P<0.001). Cluster analysis was performed using the top 50 genes with smallest P-values from the AIIG signature. As shown in Figure 4, two major branches were identified that were significantly associated with CAC status (p = 0.009, χ^2^ test). The left branch consisted of all eight men with high CAC and two men with low CAC, while the right branch consisted of six men, and all had CAC<1. Of note, the two men with low CAC on the left branch had the highest CAC scores (1.2 and 0.9) among the eight men with low-CACs. Because CAC score is highly correlated with the sums of histological artery plaque areas[10], this result suggests that the AIIG signature-atherosclerosis association exists in both genders.

### Discussion

Atherosclerosis is the main cause of clinical CVD events. Inflammation plays a central role at all stages of this disease [6]. However, the agents and associated signaling pathways responsible for initiating and perpetuating atherogenesis remain to be elucidated. The present study used global gene expression profiles of peripheral blood which identified abundant genes that are involved in innate immunity and host defense (AIIG signature) which were strongly associated with significant atherosclerosis among women with low to intermediate FRS. A similar association was also observed among men with advanced atherosclerosis who did not have history of CVD. The finding that increased expression of genes from a number of signaling pathways was associated with atherosclerosis suggests a systemic immune activation mediated through the TLR and IL-1 receptor (IL-1R) signaling pathways, and by the pro-inflammatory mediators MAP kinases (MAPK14 and FOS) and STAT3. This observation is further supported by the striking consistency of expression patterns between our and Calvano’s study, suggesting that a chronic low grade inflammation through activation of TLR/IL-1R signaling may be important in the pathogenesis of atherosclerosis, prior to the appearance of any symptoms. Thus, low grade activation of the TLR/IL-1R signaling pathway may represent a potential biomarker for use in measuring presence and/or burden of atherosclerosis.

Prior studies have demonstrated the increased expression of TLR2 and TLR4 on monocytes from patients with angina and acute coronary syndrome [11], [12]. Further, patients with recurrent unstable angina demonstrated an increased response to the TLR4 ligand lipopolysaccharide [13]. In contrast, our data suggests that the increased expression of TLR2 and TLR4, and other members of the TLR family, is observed in peripheral blood prior to the onset of clinical symptoms. Supporting the role of TLR2 and TLR4, response for peripheral blood monocytes to TLR2 and TLR4 ligands was significantly associated with the degree of coronary artery stenosis [14]. Additionally, in human atheroma, TLRs 1, 2, 4, 5 and 6 are highly up-regulated when compared to tissue from healthy arteries [15]. Further supporting the role of TLR2, employing cells isolated from carotid endarterectomies of patients with atherosclerosis, inhibition of TLR2 and MyD88, which mediates signals for all TLRs except TLR3, suppressed the expression of spontaneously expressed inflammatory mediators and matrix metalloproteinases [16].

Experimental studies have consistently demonstrated the significant role of innate immunity in atherogenesis. Genetic deletion of TLR4 or TLR2 resulted in significant reduction of arterial plaques in atherosclerosis-prone apoE or LDLR deficient mice [17], [18], [19]. Additionally, both IL1β and IL1α contribute to the pathogenesis of experimental atherosclerosis [20], [21]. Toll-like receptors have also been implicated in several autoimmune and infectious diseases [22], [23]. Epidemiological studies have demonstrated that individuals with systemic lupus erythematosus, rheumatoid arthritis, chronic infectious diseases or high levels of circulating bacterial endotoxin have a substantially increased risk of atherosclerosis [24], [25], [26], [27]. These associations may be mediated by signaling through TLR2 (which serves as a co-receptor with TLR1 or TLR6), 4, 7, 8 and 9 [28]. Each of these TLRs except TLR 7 and 9 was increased in our population with atherosclerosis.

T cell activation has been implicated in the pathogenesis of atherosclerosis. T cells are enriched in atherosclerotic lesions [29]. Further, T cell activation has been implicated in patients with acute coronary syndromes [30], [31], [32]. In contrast, the individuals with atherosclerosis in our study demonstrated a reduction in the expression of genes which contribute to T cell activation and maturation (CD3, CD3E, CD6, CD27). This apparent discrepancy may be explained by the fact that increased T cell activation has been described in patients with acute coronary syndrome. Those in our study had no angina. In fact, these observations are reminiscent of rheumatoid arthritis, which is driven mediated in large part through activation of the innate immune system [33]. Patients with rheumatoid arthritis demonstrate activated macrophages and T lymphocytes in the synovial tissue. However, these patients demonstrate decreased T cell mediated adoptive immunity in the circulation which improves following therapy [33], [34]. These observations suggest that activation of the innate immune system may result in reduction of adaptive immunity as measured peripherally.

Recently, two microarray gene expression studies [35], [36] of peripheral blood leukocytes in patients with angiographically-documented coronary stenosis were reported by Wingrove et al. and Sinnaeve et al.. We applied our innate immune gene signature to their expression data, but failed to verify the associations observed in either study. We found our 325 atherosclerosis-associated genes overlapped little (2 genes) with Sinnaeve’s 160 genes associated with presence of coronary stenosis >50%. We observed somewhat greater overlap (14 genes) between our gene signature and Wingrove’s 106 differentially-expressed gene that were identified from their literature search or their microarray results in patients referred for angiography and with ≥75% stenosis in one major coronary artery or ≥50% in two major arteries. Most of these overlapping genes belong to the TNF superfamily, IL1R signaling, and receptors for IL8 or colony stimulating factor. There are several potential reasons for the lack of substantial overlap between our results and those of Sinnaeve and Wingrove. These could include use of different measures to define the presence of atherosclerosis, since CAC presence and increased IMT may represent different (earlier) phases of atherosclerosis progression than presence of severe coronary stenosis. Likewise, there were clear differences in methods for selection of participants between studies. Our study participants were at lower risk for CVD events and are all asymptomatic, while cases in the Sinnaeve and Wingrove studies were referred for angiography on clinical grounds and some had recent acute coronary syndromes; furthermore, control participants in these two studies may have had atherosclerosis, just not severe enough to meet the case definition, whereas our control participants appear to be substantially free of atherosclerosis. Finally, there were differences in statistical analyses used to define differentially expressed genes. Sinnaeve et al used correlation analysis as opposed to two-group comparisons used in our and the Wingrove studies. All of these factors may have contributed to the minimal gene overlap observed between these 3 studies.

The findings from the present study have several potential clinical implications. Higher burden of atherosclerosis, as measured by CAC or carotid IMT, is associated with significantly greater risk for CVD events in the short term, independent of traditional risk factors [37], [38], [39], [40]. Our data suggest that several genes, especially those in the TLR/IL-1R signaling pathway, may serve as potential markers of significant atherosclerosis burden even for those predicted to be at lower risk by traditional risk equations. Toll-like receptors have been suggested as therapeutic target for several inflammatory diseases, including atherosclerosis [41]. Perhaps this signal or similar data indicating TLR/innate immune activation could be used to identify individuals who would benefit from more intensive prevention efforts. An approach that combines gene expression changes in TLR signaling and conventional CVD risk factors may enhance risk prediction for atherosclerosis. Such a strategy would require validation in larger samples.

Some limitations of the current study should be acknowledged. First, because of the cross-sectional nature of our study, the causal relationship of TLR signaling with atherosclerosis cannot be established in the present data. Second, our study only included 119 women aged 50∼86 and 16 men aged 65∼80. It is unknown whether our gene signature is applicable to identify atherosclerosis among younger adults. Larger and perspective studies are warranted to examine expression alterations of members of TLR and IL-1R signaling pathways in association with developing subclinical atherosclerosis. Nonetheless, our translational finding is unique in revealing an AIIG signature that was associated with significant atherosclerotic burden, even among a group predicted to be at low risk for atherosclerosis and clinical CVD.

### Conclusions

We used global gene expression profiles of whole blood to systematically examine the dynamic transcriptional alterations associated with atherosclerosis among women with low to intermediate predicted risk for the disease. Our results suggest the involvement of the innate immune system in atherogenesis and that whole blood provides an accessible and informative source of transcriptomal information regarding the inflammatory status of atherosclerosis in the preclinical phase of the diseases. Because of the easy access of blood samples, expression profiles of whole blood may be a useful tool to assess the burden of atherosclerosis and could potentially be used to enhance the risk prediction of CVD beyond the traditional risk factors. Future studies will be needed to validate our findings.

## Methods

### Ethics statement

The Institutional Review Boards at all Multi-Ethnic Study of Atherosclerosis (MESA) sites (Northwestern University, Wake Forest University, Johns Hopkins University, Columbia University, University of Minnesota, and UCLA) as well as the Chicago Health Aging Study (CHAS) center (Northwestern University) approved the study, and all participants gave written informed consent.

### Participants’ blood samples

A subset of 119 study participants was selected from the MESA cohort. MESA is designed to study the prevalence, risk factors, and progression of subclinical cardiovascular disease in a multiethnic cohort. A detailed description of the study design and methods has been published previously [42]. Briefly, 6814 participants aged 45 to 84 years and free of known clinical CVD were recruited from 6 US communities – Baltimore, MD, Chicago, IL, Forsyth County, NC, Los Angeles, CA, New York, NY, and St. Paul, MN. The baseline examination was performed between July 2000 and September 2002. Participants included white (38%), black (28%), Hispanic (22%), and Chinese (12%) Americans. MESA conducted three subsequent examinations of the cohort between 2002 and 2007.

In our case-control study, cases were selected as having a coronary artery calcium (CAC) score ≥100 *and* common carotid intima-media thickness (CC-IMT) ≥1.0 mm. Controls were selected as having a CAC score ≤10 and CC-IMT ≤0.65 mm. Additionally, both cases and controls were selected from those without diabetes or history of CVD who had an FRS less than 10% at baseline examination (2000∼2002). Among all MESA women, only 48 women agreed to participate and met the selection criteria as cases with significant atherosclerosis burden despite low predicted risk for CHD. We also selected potential control participants from among MESA women based on similar age and race –71 women were identified as controls and agreed to participate, giving us a ratio of 1.5 controls for each case. Out of these 119 participants, the FRS of 11 participants had increased from 11% to 20% when the MESA data for the fourth examination (2005∼2007; concurrent with the present study) became available. Carotid IMT was measured for all MESA participants only at the baseline examination. Measurement of CAC was performed for all MESA participants at the baseline examination. Follow-up testing for CAC was performed in two stages: 50% of participants returned from September 2002 to January 2004 (examination 2), and the remainder returned from March 2004 to July 2005 (examination 3). Therefore, we used the CAC score from examination 2 or 3 for these 119 participants. All these 119 participants were called back during April-July 2007 to have 5 ml whole blood drawn into two PAXgene tubes for RNA isolation.

PAXgene blood samples of additional 16 white men without diabetes or history of CVD from the CHAS cohort were selected for gene expression profiling. The blood samples of these men were collected during their clinical visits between April 2009 and July 2009. Because IMT data were not available in CHAS, cases and controls were identified based on their CAC scores. Eight of these 16 men had CAC scores 446∼1,206. The other eight had CAC scores <2. The Institutional Review Boards at each MESA site as well as the CHAS center (Chicago, IL) approved the study, and all participants gave informed consent.

### CAC and IMT Assessment

Scanning centers assessed CAC by chest-computed tomography using either a cardiac-gated electron-beam computed tomography scanner at three MESA field centers or a multi-detector computed tomography system at the other three MESA field centers. Each participant was scanned twice, and these scans were read independently at a central reading center. The amount of calcium was quantified with the Agatston scoring method. The average Agatston score was used for data analysis. Carotid IMT was measured using high-resolution B-mode ultrasonography of the right and left near and far walls of the internal carotid and common carotid arteries. Images were acquired bilaterally for both the common and internal carotid arteries. The CHAS used a similar method to assess CAC. The methodology for acquisition and interpretation of the scans has been documented previously [43], [44].

### RNA extraction and microarray experiment

Five ml whole blood from each participant was drawn into two PAXgene tubes and incubated at room temperature for three hours before being frozen at −70°C. RNAs were extracted using the PAXgene blood RNA extraction kit according to the manufacture’s protocol. The concentration of the extracted total RNA and their quality were measured by NanoDrop (Thermofisher) and Bioanalzyer 2100 (Agilent), respectively. Total RNA preparations with 260/280 ratio between 1.98–2.22 and RIN number >7.4 with enough quantity were used for the expression analysis. Globin reduction was performed using the Ambion GLOBIN clear kit. The quality of the globin-reduced RNA samples was assessed by Agilent 2100 Bioanalyzer. High quality samples were used to make first and second strand DNA followed by an IVT reaction. The size distribution of the resulting biotin labeled cRNA and the yield was checked by Agilent 2100 Bioanalyzer and NanoDrop, respectively. A normalized amount of labeled cRNA was hybridized) for 18 hours at 55°C to the Sentrix Human Ref-8 v2 Expression BeadChip for the 119 MESA samples and HumanHT-12 V4 Expression BeadChip for the 16 CHAS samples (Illumina, Inc, San Diego). After washing and staining with Cy3, the chips were scanned on the Illumina iScan. The Illumina Human Ref-8 v2 BeadChip allows genome-wide expression profiling of more than 22,000 transcripts of genes and known alternative splice variants from the RefSeq database while the HumanHT-12 V4 Expression BeadChip targets more than 47,000 transcripts of genes, gene candidates, and splice variants. The microarray analysis was performed at the Northwestern Genomic Core Facility at the Center for Genetic Medicine. All the 119 MESA RNA samples were run on 5 different days within a week. Samples collected from six MESA field centers were randomly assigned to different chips and days. All case and control samples in each MESA field center were also randomly assigned to different chips and days. The 16 CHAS RNA samples were also randomly assigned to two different chips and run on the same day.

### Quantitative RT-PCR

RNA levels of eight selected genes were analyzed by quantitative real time RT-PCR using the TaqMan chemistry on the 7300 Real-Time PCR System from Life Technologies. GAPDH was used as the endogenous control. Relative expression levels of these genes were determined by the delta-delta threshold cycle (DDC_T_) method.

### Data analysis

BeadStudio software was used to translate the scanned images into expression data, which were further log transformed and normalized by the quantile normalization procedure using the Bioconductor package: affy. Gene expression data from microarray experiments are known to have substantial background noise to signal ratios for genes expressed at the lower levels. To reduce the chance of identifying such genes, genes with a coefficient of variation less than 0.03 across all 119 MESA samples or a P value for expression detection call less than 0.01 for 60 samples or fewer were excluded, resulting in 2,057 probes for data analysis. Average linkage hierarchical clustering was performed for these 2,057 probes in the 119 MESA samples using Cluster 3.0 software (http://rana.lbl.gov/EisenSoftware.htm) using Pearson correlation as a distance metric. Significance analysis of microarray (SAM) [45] and the method developed by Storey and Tibshirani [46] were used to estimate the false discovery rate (FDR) and the number of differentially expressed genes for comparisons between case and control groups and between the two major gene expression profiling subtypes. Three widely-used applications (Ingenuity Pathway Analysis (IPA), PANTHER ontology analysis [47], and DAVID bioinformatics software [48]) were applied to identify pathways or biological functions of differentially expressed genes associated with atherosclerosis. The full MIAME-compliant microarray data were submitted to the NCBI Gene Expression Omnibus data repository (http://www.ncbi.nlm.nih.gov/geo/) under accession number GSE20129.

### Identification of major gene expression profiling subtypes

We used a multiple random validation procedure [49] to identify major gene expression profiling subtypes associated with atherosclerosis among the 119 MESA participants. Specifically, the MESA data set was first randomly divided into ten groups of approximately equal size. Each group included approximately the same number of samples from participants with significant subclinical atherosclerosis. For each group (test set), a classifier was built with the remaining nine of ten groups (training set) and then used to categorize samples in the group as atherosclerosis or non-atherosclerosis. The classification was performed across all ten groups. For each randomly drawn training set of samples, a multi-gene support vector machine classifier was constructed. For each gene, the t statistic comparing expression between cases and controls within a training set was calculated. Genes (1–100 genes) with the largest absolute values of t statistics were selected to construct a support vector machine model using an R software package, e1071. This model was then applied to classify samples within the complementary test set as atherosclerosis or non-atherosclerosis. This whole procedure was repeated 1,000 times, and the final classification status was determined by the majority vote of the 1,000 classification results (e.g. >500 votes). The samples were separated into two major gene expression profiling subtypes based on their final classification status.

### Gene selection for comparison between the present study and Calvano’s study

We used the Northwestern Unigene system to map our 2,057 Illumina probes to those in the Affymetrix U133 +2 chip used in Calvano’s study. 1,841 unique genes were found in both microarray chips.

## Supporting Information

Figure S1 Distribution of 2,057 P-values from t tests comparing all 71 controls and 48 cases among the MESA women. The dashed black line is the uniform distribution under the null hypothesis of no differential expression. If there were no differential expression observed, all the bars will be approximately at the height of the dashed black line. For these data, the observed P-value distribution is skewed to the right. The dashed red line indicates that a proportion of non-differentially expressed genes (80.4%).
(TIF)

Figure S2 Association of atherosclerosis with gene expression profiles of peripheral blood. The optimal association from the multiple random validation procedure was achieved using a set of 50–60 genes, resulting in an odds ratio of 4.85. When only the 108 women with FRS<10% were considered, the odds ratio was 5.3. SVM: support vector machine. FRS: Framingham risk score.
(TIF)

Figure S3 Multiple random validation using 50–60 genes identified 2 major gene expression molecular subtypes: 29 true positives (cases) + 17 false positives (controls); 54 true negatives (controls) + 19 false negatives (cases). Distribution of 2,057 P-values from t tests for comparisons between (a) true positives (TP, n = 29) vs false negatives (FN, n = 19) ; (b) true negatives (TN, n = 54) vs false positives (FP, n = 17); (c) TP vs FP; (d) TN vs FN. A large proportion of genes (45%) were estimated to be differentially expressed between the TPs and the FNs. Similar result was observed between the TNs and the FPs. On the other hand, no differentially expressed genes can be identified between the TPs and the FPs or between the TNs and the FNs.
(TIF)

Table S1 344 atherosclerosis-associated probes.
(DOC)

Table S2 Selected enriched pathways and functional categories.
(DOC)

Table S3 Correlation of gene expression between microarray and RT-PCR experiments.
(DOC)

Table S4 Characteristics of 16 CHAS non-diabetic white men with low or high coronary artery calcium (CAC) score.
(DOC)
