# Supplementary figures and images for: Activated TLR Signaling in Atherosclerosis among Women with Lower Framingham Risk Score: The Multi-Ethnic Study of Atherosclerosis
Source: PLoS One. 2011 Jun 16;6(6):e21067. doi: 10.1371/journal.pone.0021067 (PMC3116882; doi:10.1371/journal.pone.0021067)

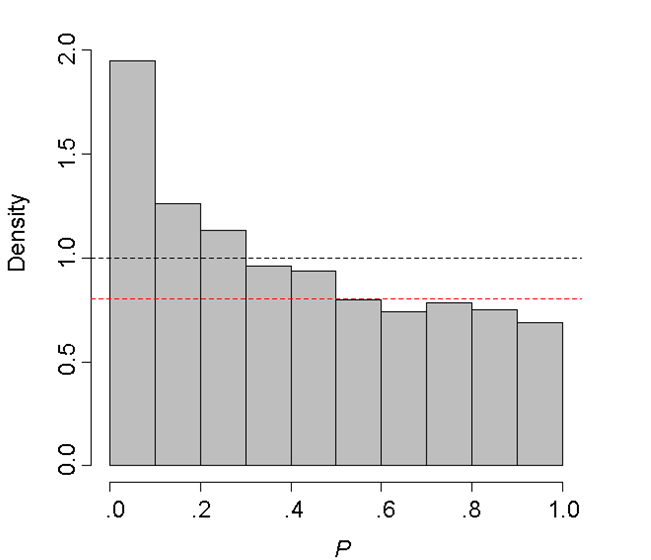

Supplement: Figure S1 — Distribution of 2,057 P-values from t tests comparing all 71 controls and 48 cases among the MESA women. The dashed black line is the uniform distribution under the null hypothesis of no differential expression. If there were no differential expression observed, all the bars will be approximately at the height of the dashed black line. For these data, the observed P-value distribution is skewed to the right. The dashed red line indicates that a proportion of non-differentially expressed genes (80.4%). (TIF) [file pone.0021067.s001.tif]

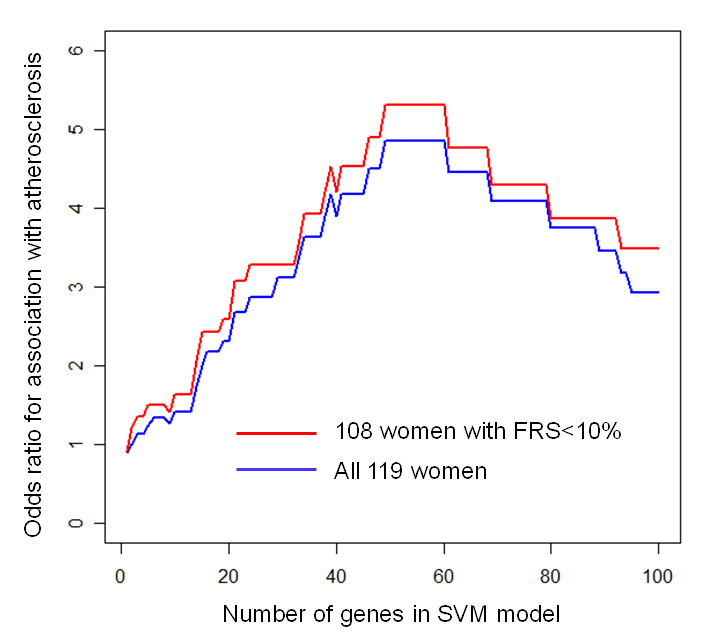

Supplement: Figure S2 — Association of atherosclerosis with gene expression profiles of peripheral blood. The optimal association from the multiple random validation procedure was achieved using a set of 50–60 genes, resulting in an odds ratio of 4.85. When only the 108 women with FRS<10% were considered, the odds ratio was 5.3. SVM: support vector machine. FRS: Framingham risk score. (TIF) [file pone.0021067.s002.tif]

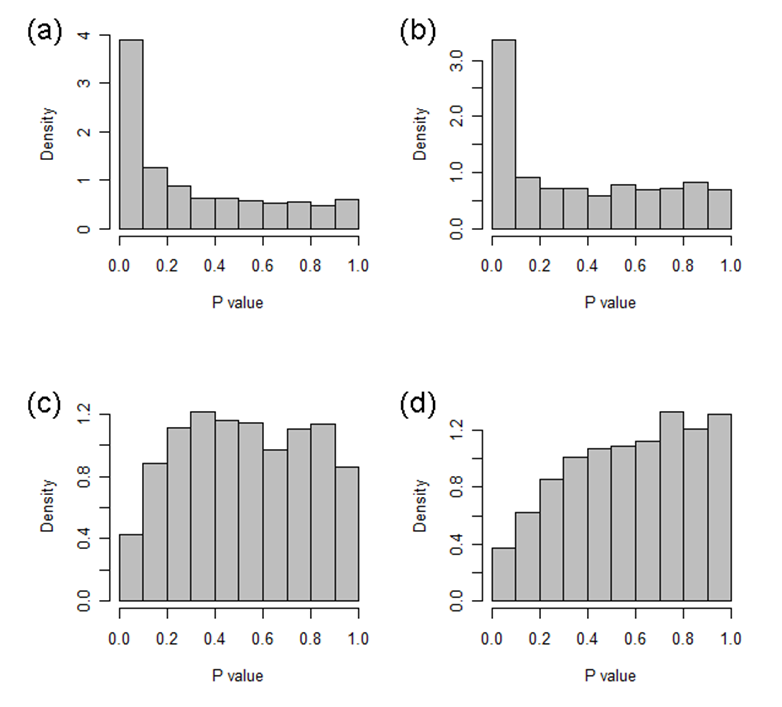

Supplement: Figure S3 — Multiple random validation using 50–60 genes identified 2 major gene expression molecular subtypes: 29 true positives (cases) + 17 false positives (controls); 54 true negatives (controls) + 19 false negatives (cases). Distribution of 2,057 P-values from t tests for comparisons between (a) true positives (TP, n = 29) vs false negatives (FN, n = 19) ; (b) true negatives (TN, n = 54) vs false positives (FP, n = 17); (c) TP vs FP; (d) TN vs FN. A large proportion of genes (45%) were estimated to be differentially expressed between the TPs and the FNs. Similar result was observed between the TNs and the FPs. On the other hand, no differentially expressed genes can be identified between the TPs and the FPs or between the TNs and the FNs. (TIF) [file pone.0021067.s003.tif]
