# Supplementary material for: Activated TLR Signaling in Atherosclerosis among Women with Lower Framingham Risk Score: The Multi-Ethnic Study of Atherosclerosis
Source: PLoS One. 2011 Jun 16;6(6):e21067. doi: 10.1371/journal.pone.0021067 (PMC3116882; doi:10.1371/journal.pone.0021067)
Supplement: Table S1 — 344 atherosclerosis-associated probes. (DOC) [file pone.0021067.s004.doc]

**Table S1: 344 atherosclerosis-associated probes**

* compare TLR-activated group with non TLR-activated group

| **Probe ID** | **Gene Symbol** | **Entrez Gene ID** | **Raw P value** | **Fold Change*** | **Gene Name** |
| --- | --- | --- | --- | --- | --- |
| ILMN_1706523 | FCGR2A | 2212 | 1.96E-12 | 1.315 | Fc fragment of IgG, low affinity IIa, receptor (CD32) |
| ILMN_1780334 | KCNJ2 | 3759 | 2.95E-11 | 1.313 | potassium inwardly-rectifying channel, subfamily J, member 2 |
| ILMN_1659544 | STX3 | 6809 | 3.08E-11 | 1.256 | syntaxin 3 |
| ILMN_1705047 | TLR8 | 51311 | 4.33E-11 | 1.288 | toll-like receptor 8 |
| ILMN_1684585 | ACSL1 | 2180 | 6.41E-11 | 1.337 | acyl-CoA synthetase long-chain family member 1 |
| ILMN_1771664 | CLEC4E | 26253 | 6.74E-11 | 1.256 | C-type lectin domain family 4, member E |
| ILMN_1657892 | TLR8 | 51311 | 7.78E-11 | 1.264 | toll-like receptor 8 |
| ILMN_1706217 | TLR4 | 7099 | 1.21E-10 | 1.305 | toll-like receptor 4 |
| ILMN_1750961 | TM6SF1 | 53346 | 2.76E-10 | 1.265 | transmembrane 6 superfamily member 1 |
| ILMN_1778059 | CASP4 | 837 | 2.83E-10 | 1.218 | caspase 4, apoptosis-related cysteine peptidase |
| ILMN_1753111 | NAMPT | 10135 | 3.99E-10 | 1.403 | nicotinamide phosphoribosyltransferase |
| ILMN_1740875 | FPRL1 | 2358 | 4.75E-10 | 1.343 | formyl peptide receptor 2 |
| ILMN_1724822 | LOC399744 | 399744 | 6.25E-10 | 1.247 | hypothetical LOC399744 |
| ILMN_1741727 | QPCT | 25797 | 6.87E-10 | 1.342 | glutaminyl-peptide cyclotransferase |
| ILMN_1710028 | MCL1 | 4170 | 7.53E-10 | 1.329 | myeloid cell leukemia sequence 1 (BCL2-related) |
| ILMN_1664525 | P2RY13 | 53829 | 1.16E-09 | 1.259 | purinergic receptor P2Y, G-protein coupled, 13 |
| ILMN_1653690 | TNFSF14 | 8740 | 1.32E-09 | 1.237 | tumor necrosis factor (ligand) superfamily, member 14 |
| ILMN_1758371 | IL1R2 | 7850 | 1.35E-09 | 1.239 | interleukin 1 receptor, type II |
| ILMN_1805175 | TGFA | 7039 | 1.38E-09 | 1.181 | transforming growth factor, alpha |
| ILMN_1719344 | LOC730820 | 730820 | 4.62E-09 | 1.238 | NA |
| ILMN_1785424 | ABLIM1 | 3983 | 4.74E-09 | 0.761 | actin binding LIM protein 1 |
| ILMN_1725471 | GK | 2710 | 4.95E-09 | 1.282 | glycerol kinase |
| ILMN_1815054 | GPR97 | 222487 | 5.36E-09 | 1.347 | G protein-coupled receptor 97 |
| ILMN_1758864 | VNN2 | 8875 | 5.85E-09 | 1.372 | vanin 2 |
| ILMN_1720771 | STX11 | 8676 | 7.67E-09 | 1.304 | syntaxin 11 |
| ILMN_1695157 | CA4 | 762 | 1.10E-08 | 1.462 | carbonic anhydrase IV |
| ILMN_1772387 | TLR2 | 7097 | 1.13E-08 | 1.232 | toll-like receptor 2 |
| ILMN_1678170 | MME | 4311 | 1.14E-08 | 1.346 | membrane metallo-endopeptidase |
| ILMN_1689046 | FLJ20273 | 54502 | 1.15E-08 | 1.224 | RNA binding motif protein 47 |
| ILMN_1714393 | RAB24 | 53917 | 1.15E-08 | 1.201 | RAB24, member RAS oncogene family |
| ILMN_1775196 | TNFRSF10B | 8795 | 1.30E-08 | 1.303 | tumor necrosis factor receptor superfamily, member 10b |
| ILMN_1688659 | IL1RN | 3557 | 1.31E-08 | 1.311 | interleukin 1 receptor antagonist |
| ILMN_1775501 | IL1B | 3553 | 1.53E-08 | 1.247 | interleukin 1, beta |
| ILMN_1779486 | FAM126B | 285172 | 1.55E-08 | 1.301 | family with sequence similarity 126, member B |
| ILMN_1749287 | TLR6 | 10333 | 1.66E-08 | 1.219 | toll-like receptor 6 |
| ILMN_1703074 | CPD | 1362 | 1.93E-08 | 1.218 | carboxypeptidase D |
| ILMN_1714643 | MGAM | 8972 | 1.94E-08 | 1.35 | maltase-glucoamylase (alpha-glucosidase) |
| ILMN_1773125 | ENTPD1 | 953 | 1.95E-08 | 1.212 | ectonucleoside triphosphate diphosphohydrolase 1 |
| ILMN_1805228 | LRG1 | 116844 | 2.00E-08 | 1.336 | leucine-rich alpha-2-glycoprotein 1 |
| ILMN_1707312 | NFIL3 | 4783 | 2.19E-08 | 1.249 | nuclear factor, interleukin 3 regulated |
| ILMN_1756725 | FFAR2 | 2867 | 2.23E-08 | 1.303 | free fatty acid receptor 2 |
| ILMN_1792521 | SMARCD3 | 6604 | 2.26E-08 | 1.243 | SWI/SNF related, matrix associated, actin dependent regulator of chromatin, subfamily d, member 3 |
| ILMN_1696466 | ROPN1L | 83853 | 2.52E-08 | 1.247 | ropporin 1-like |
| ILMN_1659753 | LAMP2 | 3920 | 2.66E-08 | 1.239 | lysosomal-associated membrane protein 2 |
| ILMN_1775542 | FAIM3 | 9214 | 2.68E-08 | 0.747 | Fas apoptotic inhibitory molecule 3 |
| ILMN_1653749 | B4GALT5 | 9334 | 3.11E-08 | 1.268 | UDP-Gal:betaGlcNAc beta 1,4- galactosyltransferase, polypeptide 5 |
| ILMN_1768761 | MYBPC3 | 4607 | 3.46E-08 | 1.205 | myosin binding protein C, cardiac |
| ILMN_1796146 | EIF4E3 | 317649 | 3.46E-08 | 1.209 | eukaryotic translation initiation factor 4E family member 3 |
| ILMN_1728677 | CREB5 | 9586 | 3.95E-08 | 1.401 | cAMP responsive element binding protein 5 |
| ILMN_1687960 | LIMK2 | 3985 | 4.14E-08 | 1.243 | LIM domain kinase 2 |
| ILMN_1798475 | CSF2RB | 1439 | 4.29E-08 | 1.179 | colony stimulating factor 2 receptor, beta, low-affinity (granulocyte-macrophage) |
| ILMN_1726545 | LILRA5 | 353514 | 4.64E-08 | 1.234 | leukocyte immunoglobulin-like receptor, subfamily A (with TM domain), member 5 |
| ILMN_1788002 | MAPK14 | 1432 | 4.67E-08 | 1.188 | mitogen-activated protein kinase 14 |
| ILMN_1675756 | KCNJ15 | 3772 | 4.73E-08 | 1.325 | potassium inwardly-rectifying channel, subfamily J, member 15 |
| ILMN_1772036 | STEAP4 | 79689 | 5.01E-08 | 1.209 | STEAP family member 4 |
| ILMN_1757361 | NCF4 | 4689 | 5.67E-08 | 1.179 | neutrophil cytosolic factor 4, 40kDa |
| ILMN_1757074 | GNG10 | 2790 | 5.82E-08 | 1.343 | guanine nucleotide binding protein (G protein), gamma 10 |
| ILMN_1663753 | PILRA | 29992 | 5.84E-08 | 1.278 | paired immunoglobin-like type 2 receptor alpha |
| ILMN_1675424 | SIRPB1 | 10326 | 6.36E-08 | 1.26 | signal-regulatory protein beta 1 |
| ILMN_1651942 | CDC25B | 994 | 8.87E-08 | 0.808 | cell division cycle 25 homolog B (S. pombe) |
| ILMN_1736180 | FRAT1 | 10023 | 9.17E-08 | 1.228 | frequently rearranged in advanced T-cell lymphomas |
| ILMN_1799765 | RAB24 | 53917 | 1.00E-07 | 1.195 | RAB24, member RAS oncogene family |
| ILMN_1743570 | CEACAM3 | 1084 | 1.00E-07 | 1.25 | carcinoembryonic antigen-related cell adhesion molecule 3 |
| ILMN_1785005 | NCF4 | 4689 | 1.04E-07 | 1.217 | neutrophil cytosolic factor 4, 40kDa |
| ILMN_1731048 | TLR1 | 7096 | 1.30E-07 | 1.226 | toll-like receptor 1 |
| ILMN_1798021 | CCDC64 | 92558 | 1.64E-07 | 0.839 | coiled-coil domain containing 64 |
| ILMN_1804738 | MEFV | 4210 | 1.69E-07 | 1.198 | Mediterranean fever |
| ILMN_1799106 | MOSC1 | 64757 | 1.86E-07 | 1.343 | MOCO sulphurase C-terminal domain containing 1 |
| ILMN_1687519 | SNAP23 | 8773 | 1.92E-07 | 1.192 | synaptosomal-associated protein, 23kDa |
| ILMN_1697493 | WDFY3 | 23001 | 2.29E-07 | 1.214 | WD repeat and FYVE domain containing 3 |
| ILMN_1665964 | GAB2 | 9846 | 2.39E-07 | 1.237 | GRB2-associated binding protein 2 |
| ILMN_1655702 | ABHD5 | 51099 | 2.68E-07 | 1.218 | abhydrolase domain containing 5 |
| ILMN_1749892 | EGLN1 | 54583 | 2.71E-07 | 1.197 | egl nine homolog 1 (C. elegans) |
| ILMN_1718982 | BEST1 | 7439 | 2.99E-07 | 1.256 | bestrophin 1 |
| ILMN_1808047 | PHC2 | 1912 | 3.15E-07 | 1.267 | polyhomeotic homolog 2 (Drosophila) |
| ILMN_1807649 | LOC339745 | 339745 | 3.22E-07 | 1.173 | speckle-type POZ protein-like |
| ILMN_1696394 | IL6R | 3570 | 3.36E-07 | 1.225 | interleukin 6 receptor |
| ILMN_1778968 | ZNF438 | 220929 | 3.39E-07 | 1.166 | zinc finger protein 438 |
| ILMN_1731188 | KBTBD7 | 84078 | 3.66E-07 | 1.183 | kelch repeat and BTB (POZ) domain containing 7 |
| ILMN_1781416 | FRAT1 | 10023 | 3.68E-07 | 1.248 | frequently rearranged in advanced T-cell lymphomas |
| ILMN_1700850 | PLXNC1 | 10154 | 3.96E-07 | 1.239 | plexin C1 |
| ILMN_1703108 | UBE2L6 | 9246 | 4.03E-07 | 1.257 | ubiquitin-conjugating enzyme E2L 6 |
| ILMN_1667985 | NTNG2 | 84628 | 4.42E-07 | 1.232 | netrin G2 |
| ILMN_1688959 | CD27 | 939 | 5.08E-07 | 0.795 | CD27 molecule |
| ILMN_1809850 | RCN3 | 57333 | 5.58E-07 | 1.173 | reticulocalbin 3, EF-hand calcium binding domain |
| ILMN_1809173 | LOC729021 | 729021 | 5.69E-07 | 1.257 | hypothetical protein LOC729021 |
| ILMN_1653871 | NAMPT | 10135 | 6.27E-07 | 1.291 | nicotinamide phosphoribosyltransferase |
| ILMN_1732923 | SIPA1L2 | 57568 | 6.66E-07 | 1.271 | signal-induced proliferation-associated 1 like 2 |
| ILMN_1744508 | FAM53C | 51307 | 7.06E-07 | 1.192 | family with sequence similarity 53, member C |
| ILMN_1719986 | PIK3IP1 | 113791 | 7.24E-07 | 0.778 | phosphoinositide-3-kinase interacting protein 1 |
| ILMN_1745262 | REM2 | 161253 | 7.96E-07 | 1.262 | RAS (RAD and GEM)-like GTP binding 2 |
| ILMN_1746436 | HCG27 | 253018 | 7.98E-07 | 1.239 | HLA complex group 27 |
| ILMN_1798977 | GPR97 | 222487 | 9.11E-07 | 1.24 | G protein-coupled receptor 97 |
| ILMN_1700610 | CLEC7A | 64581 | 9.52E-07 | 1.213 | C-type lectin domain family 7, member A |
| ILMN_1794875 | MAG1 | 84803 | 9.87E-07 | 1.261 | 1-acylglycerol-3-phosphate O-acyltransferase 9 |
| ILMN_1652490 | MANSC1 | 54682 | 1.01E-06 | 1.247 | MANSC domain containing 1 |
| ILMN_1755873 | EMR3 | 84658 | 1.07E-06 | 1.216 | egf-like module containing, mucin-like, hormone receptor-like 3 |
| ILMN_1679268 | PELI1 | 57162 | 1.17E-06 | 1.196 | pellino homolog 1 (Drosophila) |
| ILMN_1665943 | MAP4K1 | 11184 | 1.23E-06 | 0.834 | mitogen-activated protein kinase kinase kinase kinase 1 |
| ILMN_1783085 | IL8RB | 3579 | 1.29E-06 | 1.24 | interleukin 8 receptor, beta |
| ILMN_1789019 | CSF3R | 1441 | 1.39E-06 | 1.257 | colony stimulating factor 3 receptor (granulocyte) |
| ILMN_1679196 | KIAA0125 | 9834 | 1.41E-06 | 0.841 | KIAA0125 |
| ILMN_1810420 | DYSF | 8291 | 1.46E-06 | 1.36 | dysferlin, limb girdle muscular dystrophy 2B (autosomal recessive) |
| ILMN_1731714 | CREB5 | 9586 | 1.47E-06 | 1.264 | cAMP responsive element binding protein 5 |
| ILMN_1723486 | HK2 | 3099 | 1.50E-06 | 1.172 | hexokinase 2 |
| ILMN_1701837 | KLHL2 | 11275 | 1.50E-06 | 1.22 | kelch-like 2, Mayven (Drosophila) |
| ILMN_1651429 | SELM | 140606 | 1.53E-06 | 0.853 | selenoprotein M |
| ILMN_1768980 | ST8SIA4 | 7903 | 1.67E-06 | 1.166 | ST8 alpha-N-acetyl-neuraminide alpha-2,8-sialyltransferase 4 |
| ILMN_1809040 | LDLRAP1 | 26119 | 1.71E-06 | 0.849 | low density lipoprotein receptor adaptor protein 1 |
| ILMN_1772131 | IL1R2 | 7850 | 1.78E-06 | 1.286 | interleukin 1 receptor, type II |
| ILMN_1691508 | PLAUR | 5329 | 1.83E-06 | 1.206 | plasminogen activator, urokinase receptor |
| ILMN_1779426 | RAB11FIP1 | 80223 | 1.88E-06 | 1.172 | RAB11 family interacting protein 1 (class I) |
| ILMN_1736048 | ELL | 8178 | 1.89E-06 | 1.209 | elongation factor RNA polymerase II |
| ILMN_1673111 | TSEN34 | 79042 | 1.91E-06 | 1.212 | tRNA splicing endonuclease 34 homolog (S. cerevisiae) |
| ILMN_1798448 | IDS | 3423 | 2.02E-06 | 1.158 | iduronate 2-sulfatase |
| ILMN_1780546 | OSM | 5008 | 2.13E-06 | 1.166 | oncostatin M |
| ILMN_1809522 | BTBD14A | 138151 | 2.14E-06 | 1.174 | NACC family member 2, BEN and BTB (POZ) domain containing |
| ILMN_1749078 | TIMP2 | 7077 | 2.18E-06 | 1.184 | TIMP metallopeptidase inhibitor 2 |
| ILMN_1717594 | DKFZp761E198 | 91056 | 2.21E-06 | 1.263 | DKFZp761E198 protein |
| ILMN_1697309 | NCF1 | 653361 | 2.46E-06 | 1.258 | neutrophil cytosolic factor 1 |
| ILMN_1737627 | MAPK14 | 1432 | 2.62E-06 | 1.159 | mitogen-activated protein kinase 14 |
| ILMN_1666507 | PLAUR | 5329 | 2.95E-06 | 1.225 | plasminogen activator, urokinase receptor |
| ILMN_1766094 | MOSPD2 | 158747 | 2.98E-06 | 1.179 | motile sperm domain containing 2 |
| ILMN_1782070 | NPL | 80896 | 3.02E-06 | 1.194 | N-acetylneuraminate pyruvate lyase (dihydrodipicolinate synthase) |
| ILMN_1752455 | DOCK5 | 80005 | 3.05E-06 | 1.162 | dedicator of cytokinesis 5 |
| ILMN_1701402 | IKIP | 121457 | 3.18E-06 | 1.161 | IKK interacting protein |
| ILMN_1750158 | ACOX1 | 51 | 3.23E-06 | 1.253 | acyl-Coenzyme A oxidase 1, palmitoyl |
| ILMN_1737964 | HIATL1 | 84641 | 3.28E-06 | 1.189 | hippocampus abundant transcript-like 1 |
| ILMN_1668270 | ZDHHC18 | 84243 | 3.51E-06 | 1.216 | zinc finger, DHHC-type containing 18 |
| ILMN_1729915 | PILRA | 29992 | 3.51E-06 | 1.218 | paired immunoglobin-like type 2 receptor alpha |
| ILMN_1656042 | KIAA0319L | 79932 | 3.58E-06 | 1.19 | KIAA0319-like |
| ILMN_1691119 | RNF122 | 79845 | 3.63E-06 | 1.155 | ring finger protein 122 |
| ILMN_1749673 | CSF2RA | 1438 | 3.67E-06 | 1.174 | colony stimulating factor 2 receptor, alpha, low-affinity (granulocyte-macrophage) |
| ILMN_1661695 | IRAK3 | 11213 | 4.03E-06 | 1.187 | interleukin-1 receptor-associated kinase 3 |
| ILMN_1662524 | IL8RA | 3577 | 4.08E-06 | 1.247 | interleukin 8 receptor, alpha |
| ILMN_1750086 | ST3GAL4 | 6484 | 4.10E-06 | 1.23 | ST3 beta-galactoside alpha-2,3-sialyltransferase 4 |
| ILMN_1677511 | PTGS2 | 5743 | 4.11E-06 | 1.18 | prostaglandin-endoperoxide synthase 2 (prostaglandin G/H synthase and cyclooxygenase) |
| ILMN_1775522 | MAGED1 | 9500 | 4.15E-06 | 0.849 | melanoma antigen family D, 1 |
| ILMN_1712431 | FAM113B | 91523 | 4.29E-06 | 0.823 | family with sequence similarity 113, member B |
| ILMN_1658706 | ST6GALNAC2 | 10610 | 4.45E-06 | 1.249 | ST6 (alpha-N-acetyl-neuraminyl-2,3-beta-galactosyl-1,3)-N-acetylgalactosaminide alpha-2,6-sialyltransferase 2 |
| ILMN_1804743 | USP32 | 84669 | 4.55E-06 | 1.17 | ubiquitin specific peptidase 32 |
| ILMN_1743933 | TSHZ3 | 57616 | 4.90E-06 | 1.205 | teashirt zinc finger homeobox 3 |
| ILMN_1685057 | SLC22A4 | 6583 | 4.93E-06 | 1.185 | solute carrier family 22 (organic cation/ergothioneine transporter), member 4 |
| ILMN_1679339 | C3orf62 | 375341 | 5.33E-06 | 1.154 | chromosome 3 open reading frame 62 |
| ILMN_1765880 | C16orf57 | 79650 | 5.41E-06 | 1.205 | chromosome 16 open reading frame 57 |
| ILMN_1797822 | KIAA0746 | 23231 | 5.95E-06 | 0.834 | KIAA0746 protein |
| ILMN_1785095 | ATP6V0E2 | 155066 | 6.69E-06 | 0.817 | ATPase, H+ transporting V0 subunit e2 |
| ILMN_1722981 | TLR5 | 7100 | 6.85E-06 | 1.249 | toll-like receptor 5 |
| ILMN_1801767 | ABHD3 | 171586 | 6.98E-06 | 1.205 | abhydrolase domain containing 3 |
| ILMN_1708934 | ADM | 133 | 8.37E-06 | 1.268 | adrenomedullin |
| ILMN_1736976 | HPCAL4 | 51440 | 8.40E-06 | 0.831 | hippocalcin like 4 |
| ILMN_1688373 | LST1 | 7940 | 8.57E-06 | 1.153 | leukocyte specific transcript 1 |
| ILMN_1756937 | ST8SIA4 | 7903 | 8.93E-06 | 1.166 | ST8 alpha-N-acetyl-neuraminide alpha-2,8-sialyltransferase 4 |
| ILMN_1663618 | STAT3 | 6774 | 8.97E-06 | 1.189 | signal transducer and activator of transcription 3 (acute-phase response factor) |
| ILMN_1793743 | DIRC2 | 84925 | 9.20E-06 | 1.163 | disrupted in renal carcinoma 2 |
| ILMN_1758963 | NADK | 65220 | 9.27E-06 | 1.201 | NAD kinase |
| ILMN_1781285 | DUSP1 | 1843 | 9.47E-06 | 1.201 | dual specificity phosphatase 1 |
| ILMN_1690112 | LAT2 | 7462 | 9.85E-06 | 1.162 | linker for activation of T cells family, member 2 |
| ILMN_1655414 | TNFSF14 | 8740 | 9.89E-06 | 1.205 | tumor necrosis factor (ligand) superfamily, member 14 |
| ILMN_1781102 | C14orf138 | 79609 | 1.11E-05 | 1.162 | chromosome 14 open reading frame 138 |
| ILMN_1694548 | ANXA3 | 306 | 1.14E-05 | 1.232 | annexin A3 |
| ILMN_1672417 | PTPRCAP | 5790 | 1.16E-05 | 0.846 | protein tyrosine phosphatase, receptor type, C-associated protein |
| ILMN_1740160 | PLCG1 | 5335 | 1.19E-05 | 0.856 | phospholipase C, gamma 1 |
| ILMN_1674394 | C20orf3 | 57136 | 1.20E-05 | 1.198 | chromosome 20 open reading frame 3 |
| ILMN_1677693 | GPR109B | 8843 | 1.20E-05 | 1.201 | niacin receptor 2 |
| ILMN_1741881 | C9orf72 | 203228 | 1.21E-05 | 1.168 | chromosome 9 open reading frame 72 |
| ILMN_1806863 | DGKA | 1606 | 1.22E-05 | 0.859 | diacylglycerol kinase, alpha 80kDa |
| ILMN_1692742 | DENND3 | 22898 | 1.24E-05 | 1.165 | DENN/MADD domain containing 3 |
| ILMN_1766425 | REPS2 | 9185 | 1.25E-05 | 1.202 | RALBP1 associated Eps domain containing 2 |
| ILMN_1696187 | PYGL | 5836 | 1.27E-05 | 1.188 | phosphorylase, glycogen, liver |
| ILMN_1746836 | PTAFR | 5724 | 1.30E-05 | 1.215 | platelet-activating factor receptor |
| ILMN_1657129 | SKAP2 | 8935 | 1.32E-05 | 1.194 | src kinase associated phosphoprotein 2 |
| ILMN_1712577 | TMEM157 | 345757 | 1.33E-05 | 1.164 | family with sequence similarity 174, member A |
| ILMN_1745112 | FAM102A | 399665 | 1.43E-05 | 0.875 | family with sequence similarity 102, member A |
| ILMN_1812721 | LOC728014 | 728014 | 1.47E-05 | 0.831 | NA |
| ILMN_1803984 | MAK | 4117 | 1.48E-05 | 1.189 | male germ cell-associated kinase |
| ILMN_1750497 | GPR109A | 338442 | 1.50E-05 | 1.217 | niacin receptor 1 |
| ILMN_1812618 | CENTD3 | 64411 | 1.50E-05 | 1.201 | ArfGAP with RhoGAP domain, ankyrin repeat and PH domain 3 |
| ILMN_1674160 | BIN1 | 274 | 1.53E-05 | 0.809 | bridging integrator 1 |
| ILMN_1753312 | PLXDC2 | 84898 | 1.57E-05 | 1.176 | plexin domain containing 2 |
| ILMN_1694810 | PANX2 | 56666 | 1.58E-05 | 1.278 | pannexin 2 |
| ILMN_1685312 | PSG3 | 5671 | 1.63E-05 | 1.18 | pregnancy specific beta-1-glycoprotein 3 |
| ILMN_1652143 | E2F3 | 1871 | 1.68E-05 | 1.179 | E2F transcription factor 3 |
| ILMN_1692023 | SIPA1L1 | 26037 | 1.82E-05 | 1.183 | signal-induced proliferation-associated 1 like 1 |
| ILMN_1787127 | SLC43A2 | 124935 | 1.86E-05 | 1.207 | solute carrier family 43, member 2 |
| ILMN_1717207 | MMP25 | 64386 | 1.89E-05 | 1.282 | matrix metallopeptidase 25 |
| ILMN_1724666 | INADL | 10207 | 1.92E-05 | 0.868 | InaD-like (Drosophila) |
| ILMN_1678928 | SLCO3A1 | 28232 | 1.93E-05 | 1.152 | solute carrier organic anion transporter family, member 3A1 |
| ILMN_1763809 | IRS2 | 8660 | 2.00E-05 | 1.206 | insulin receptor substrate 2 |
| ILMN_1665761 | BCL11B | 64919 | 2.07E-05 | 0.833 | B-cell CLL/lymphoma 11B (zinc finger protein) |
| ILMN_1659255 | RP2 | 6102 | 2.17E-05 | 1.153 | retinitis pigmentosa 2 (X-linked recessive) |
| ILMN_1662741 | EDG4 | 9170 | 2.19E-05 | 1.192 | lysophosphatidic acid receptor 2 |
| ILMN_1806165 | HSPA6 | 3310 | 2.45E-05 | 1.183 | heat shock 70kDa protein 6 (HSP70B') |
| ILMN_1754753 | IL6R | 3570 | 2.59E-05 | 1.204 | interleukin 6 receptor |
| ILMN_1688231 | TREM1 | 54210 | 2.60E-05 | 1.248 | triggering receptor expressed on myeloid cells 1 |
| ILMN_1787749 | CASP8 | 841 | 2.61E-05 | 1.137 | caspase 8, apoptosis-related cysteine peptidase |
| ILMN_1732575 | SEC14L1 | 6397 | 2.63E-05 | 1.164 | SEC14-like 1 (S. cerevisiae) |
| ILMN_1741482 | GNAQ | 2776 | 2.72E-05 | 1.168 | guanine nucleotide binding protein (G protein), q polypeptide |
| ILMN_1663422 | Rgr | 266747 | 2.89E-05 | 1.205 | ral guanine nucleotide dissociation stimulator-like 4 |
| ILMN_1749868 | C10orf38 | 221061 | 2.98E-05 | 0.889 | family with sequence similarity 171, member A1 |
| ILMN_1814194 | TCF4 | 6925 | 3.10E-05 | 0.86 | transcription factor 4 |
| ILMN_1757730 | TTC27 | 55622 | 3.11E-05 | 0.885 | tetratricopeptide repeat domain 27 |
| ILMN_1799644 | IBRDC2 | 255488 | 3.14E-05 | 1.14 | ring finger protein 144B |
| ILMN_1752932 | EVA1 | 10205 | 3.18E-05 | 1.224 | myelin protein zero-like 2 |
| ILMN_1663526 | SPTAN1 | 6709 | 3.33E-05 | 0.848 | spectrin, alpha, non-erythrocytic 1 (alpha-fodrin) |
| ILMN_1790689 | CRISPLD2 | 83716 | 3.34E-05 | 1.287 | cysteine-rich secretory protein LCCL domain containing 2 |
| ILMN_1728478 | CXCL16 | 58191 | 3.42E-05 | 1.208 | chemokine (C-X-C motif) ligand 16 |
| ILMN_1685493 | TMCC3 | 57458 | 3.49E-05 | 1.17 | transmembrane and coiled-coil domain family 3 |
| ILMN_1672398 | RAB20 | 55647 | 3.50E-05 | 1.239 | RAB20, member RAS oncogene family |
| ILMN_1761260 | COBLL1 | 22837 | 3.60E-05 | 0.823 | COBL-like 1 |
| ILMN_1720270 | CDR2 | 1039 | 3.83E-05 | 0.861 | cerebellar degeneration-related protein 2, 62kDa |
| ILMN_1671568 | ECHDC2 | 55268 | 3.95E-05 | 0.847 | enoyl Coenzyme A hydratase domain containing 2 |
| ILMN_1785570 | SUSD3 | 203328 | 4.21E-05 | 0.849 | sushi domain containing 3 |
| ILMN_1769383 | GIMAP5 | 55340 | 4.24E-05 | 0.829 | GTPase, IMAP family member 5 |
| ILMN_1747759 | WSB1 | 26118 | 4.26E-05 | 1.153 | WD repeat and SOCS box-containing 1 |
| ILMN_1804935 | VNN3 | 55350 | 4.27E-05 | 1.23 | vanin 3 |
| ILMN_1733221 | C9orf164 | 349236 | 4.58E-05 | 1.148 | NA |
| ILMN_1770299 | RAB11FIP1 | 80223 | 5.06E-05 | 1.164 | RAB11 family interacting protein 1 (class I) |
| ILMN_1731275 | DPEP3 | 64180 | 5.11E-05 | 1.203 | dipeptidase 3 |
| ILMN_1808059 | BCAS4 | 55653 | 5.18E-05 | 0.857 | breast carcinoma amplified sequence 4 |
| ILMN_1667994 | AMD1 | 262 | 5.24E-05 | 1.132 | adenosylmethionine decarboxylase 1 |
| ILMN_1680453 | ITM2C | 81618 | 5.30E-05 | 0.848 | integral membrane protein 2C |
| ILMN_1671260 | GPR177 | 79971 | 5.42E-05 | 1.281 | G protein-coupled receptor 177 |
| ILMN_1762284 | SASP | 151516 | 5.62E-05 | 1.181 | aspartic peptidase, retroviral-like 1 |
| ILMN_1751400 | SKAP1 | 8631 | 5.75E-05 | 0.853 | src kinase associated phosphoprotein 1 |
| ILMN_1745256 | CXXC5 | 51523 | 5.93E-05 | 0.855 | CXXC finger 5 |
| ILMN_1696003 | GNAI3 | 2773 | 6.10E-05 | 1.141 | guanine nucleotide binding protein (G protein), alpha inhibiting activity polypeptide 3 |
| ILMN_1812278 | LY9 | 4063 | 6.26E-05 | 0.875 | lymphocyte antigen 9 |
| ILMN_1699772 | RRAGD | 58528 | 6.27E-05 | 1.127 | Ras-related GTP binding D |
| ILMN_1690625 | SLC9A8 | 23315 | 6.60E-05 | 1.143 | solute carrier family 9 (sodium/hydrogen exchanger), member 8 |
| ILMN_1715131 | CCR7 | 1236 | 6.65E-05 | 0.76 | chemokine (C-C motif) receptor 7 |
| ILMN_1770641 | KLHL3 | 26249 | 6.66E-05 | 0.87 | kelch-like 3 (Drosophila) |
| ILMN_1813769 | ARRDC3 | 57561 | 6.67E-05 | 1.132 | arrestin domain containing 3 |
| ILMN_1689836 | C5AR1 | 728 | 6.70E-05 | 1.212 | complement component 5a receptor 1 |
| ILMN_1728639 | FCGR3B | 2215 | 6.82E-05 | 1.227 | Fc fragment of IgG, low affinity IIIb, receptor (CD16b) |
| ILMN_1716360 | CLIC1 | 1192 | 7.09E-05 | 1.127 | chloride intracellular channel 1 |
| ILMN_1815500 | ITPR3 | 3710 | 7.19E-05 | 0.839 | inositol 1,4,5-triphosphate receptor, type 3 |
| ILMN_1806999 | MGC4093 | 80776 | 7.25E-05 | 1.138 | B9 protein domain 2 |
| ILMN_1665217 | C3orf34 | 84984 | 7.30E-05 | 1.152 | chromosome 3 open reading frame 34 |
| ILMN_1664644 | ATG16L2 | 89849 | 7.42E-05 | 1.185 | ATG16 autophagy related 16-like 2 (S. cerevisiae) |
| ILMN_1682368 | DKFZp434K1815 | 222229 | 7.46E-05 | 1.182 | leucine-rich repeats and WD repeat domain containing 1 |
| ILMN_1676665 | CUEDC1 | 404093 | 7.48E-05 | 1.171 | CUE domain containing 1 |
| ILMN_1727045 | RASGRP3 | 25780 | 7.58E-05 | 0.843 | RAS guanyl releasing protein 3 (calcium and DAG-regulated) |
| ILMN_1713058 | PSTPIP2 | 9050 | 7.86E-05 | 1.156 | proline-serine-threonine phosphatase interacting protein 2 |
| ILMN_1775257 | PROK2 | 60675 | 7.92E-05 | 1.25 | prokineticin 2 |
| ILMN_1807529 | PADI4 | 23569 | 7.97E-05 | 1.253 | peptidyl arginine deiminase, type IV |
| ILMN_1779015 | ZNF467 | 168544 | 8.51E-05 | 1.204 | zinc finger protein 467 |
| ILMN_1748694 | LOC340527 | 340527 | 8.57E-05 | 1.225 | NHS-like 2 |
| ILMN_1670130 | ARID3A | 1820 | 8.90E-05 | 1.173 | AT rich interactive domain 3A (BRIGHT-like) |
| ILMN_1796349 | SMPDL3A | 10924 | 8.92E-05 | 1.147 | sphingomyelin phosphodiesterase, acid-like 3A |
| ILMN_1677466 | DUSP6 | 1848 | 9.03E-05 | 1.155 | dual specificity phosphatase 6 |
| ILMN_1755555 | BLR1 | 643 | 0.000104015 | 0.783 | chemokine (C-X-C motif) receptor 5 |
| ILMN_1719998 | C9orf45 | NA | 0.000106394 | 0.817 | NA |
| ILMN_1684402 | STXBP5 | 134957 | 0.000109619 | 1.137 | syntaxin binding protein 5 (tomosyn) |
| ILMN_1761566 | C5orf32 | 84418 | 0.000116916 | 1.224 | chromosome 5 open reading frame 32 |
| ILMN_1662451 | FCER2 | 2208 | 0.000116941 | 0.836 | Fc fragment of IgE, low affinity II, receptor for (CD23) |
| ILMN_1706825 | PKN2 | 5586 | 0.000117698 | 1.131 | protein kinase N2 |
| ILMN_1709233 | F5 | 2153 | 0.000124418 | 1.174 | coagulation factor V (proaccelerin, labile factor) |
| ILMN_1717552 | MAN2C1 | 4123 | 0.000125696 | 0.876 | mannosidase, alpha, class 2C, member 1 |
| ILMN_1787843 | HSDL2 | 84263 | 0.000126103 | 1.113 | hydroxysteroid dehydrogenase like 2 |
| ILMN_1702301 | DOCK10 | 55619 | 0.00012623 | 0.862 | dedicator of cytokinesis 10 |
| ILMN_1699160 | ITK | 3702 | 0.000128786 | 0.845 | IL2-inducible T-cell kinase |
| ILMN_1719756 | ZAP70 | 7535 | 0.00013156 | 0.878 | zeta-chain (TCR) associated protein kinase 70kDa |
| ILMN_1815010 | RNF141 | 50862 | 0.00013267 | 1.114 | ring finger protein 141 |
| ILMN_1732296 | ID3 | 3399 | 0.000137311 | 0.859 | inhibitor of DNA binding 3, dominant negative helix-loop-helix protein |
| ILMN_1729161 | NOTCH1 | 4851 | 0.000139494 | 1.177 | Notch homolog 1, translocation-associated (Drosophila) |
| ILMN_1775235 | AFF3 | 3899 | 0.000141347 | 0.833 | AF4/FMR2 family, member 3 |
| ILMN_1670054 | TIMP2 | 7077 | 0.000142339 | 1.165 | TIMP metallopeptidase inhibitor 2 |
| ILMN_1782125 | DSC2 | 1824 | 0.000146442 | 1.271 | desmocollin 2 |
| ILMN_1774077 | GBP2 | 2634 | 0.000150483 | 1.203 | guanylate binding protein 2, interferon-inducible |
| ILMN_1661361 | WWC3 | 55841 | 0.000151901 | 1.157 | WWC family member 3 |
| ILMN_1701603 | ALPL | 249 | 0.000153021 | 1.342 | alkaline phosphatase, liver/bone/kidney |
| ILMN_1659227 | CD79A | 973 | 0.000153137 | 0.813 | CD79a molecule, immunoglobulin-associated alpha |
| ILMN_1721563 | TMEM127 | 55654 | 0.000157819 | 1.125 | transmembrane protein 127 |
| ILMN_1798270 | C11orf75 | 56935 | 0.000160531 | 1.17 | chromosome 11 open reading frame 75 |
| ILMN_1736190 | CYP4F3 | 4051 | 0.000161769 | 1.249 | cytochrome P450, family 4, subfamily F, polypeptide 3 |
| ILMN_1758735 | NLRP12 | 91662 | 0.00016239 | 1.166 | NLR family, pyrin domain containing 12 |
| ILMN_1679797 | ADARB1 | 104 | 0.000169869 | 0.872 | adenosine deaminase, RNA-specific, B1 (RED1 homolog rat) |
| ILMN_1664068 | ERGIC1 | 57222 | 0.000177585 | 1.228 | endoplasmic reticulum-golgi intermediate compartment (ERGIC) 1 |
| ILMN_1731224 | PARP9 | 83666 | 0.000178888 | 1.174 | poly (ADP-ribose) polymerase family, member 9 |
| ILMN_1746565 | CD6 | 923 | 0.000181067 | 0.843 | CD6 molecule |
| ILMN_1684440 | PXN | 5829 | 0.000186407 | 1.127 | paxillin |
| ILMN_1716815 | CEACAM1 | 634 | 0.000187582 | 1.21 | carcinoembryonic antigen-related cell adhesion molecule 1 (biliary glycoprotein) |
| ILMN_1759401 | PISD | 23761 | 0.000192072 | 1.161 | phosphatidylserine decarboxylase |
| ILMN_1658494 | C13orf15 | 28984 | 0.000192129 | 0.837 | chromosome 13 open reading frame 15 |
| ILMN_1744137 | LOC728776 | 728776 | 0.000192714 | 0.855 | high-mobility group nucleosome binding domain 1 pseudogene |
| ILMN_1665557 | USP15 | 9958 | 0.000194806 | 1.154 | ubiquitin specific peptidase 15 |
| ILMN_1704870 | PGLYRP1 | 8993 | 0.000205634 | 1.294 | peptidoglycan recognition protein 1 |
| ILMN_1668277 | BLK | 640 | 0.000205849 | 0.82 | B lymphoid tyrosine kinase |
| ILMN_1656203 | RPS8 | 6202 | 0.00020847 | 0.824 | ribosomal protein S8 |
| ILMN_1742126 | CDCA7L | 55536 | 0.00021099 | 0.815 | cell division cycle associated 7-like |
| ILMN_1700147 | VPREB3 | 29802 | 0.000212749 | 0.773 | pre-B lymphocyte 3 |
| ILMN_1673757 | CASP8 | 841 | 0.000212749 | 1.15 | caspase 8, apoptosis-related cysteine peptidase |
| ILMN_1679162 | TNFAIP2 | 7127 | 0.000213634 | 1.168 | tumor necrosis factor, alpha-induced protein 2 |
| ILMN_1747506 | DHX34 | 9704 | 0.000214492 | 1.192 | DEAH (Asp-Glu-Ala-His) box polypeptide 34 |
| ILMN_1721116 | USP10 | 9100 | 0.000218251 | 1.204 | ubiquitin specific peptidase 10 |
| ILMN_1656361 | LOC201175 | 201175 | 0.000222138 | 1.189 | SH3 domain containing 20 |
| ILMN_1748915 | S100A12 | 6283 | 0.000227365 | 1.221 | S100 calcium binding protein A12 |
| ILMN_1716928 | TLE3 | 7090 | 0.000228054 | 1.202 | transducin-like enhancer of split 3 (E(sp1) homolog, Drosophila) |
| ILMN_1695079 | ZNF101 | 94039 | 0.000228316 | 0.899 | zinc finger protein 101 |
| ILMN_1679185 | LEF1 | 51176 | 0.000229911 | 0.799 | lymphoid enhancer-binding factor 1 |
| ILMN_1695985 | OAZ2 | 4947 | 0.000233758 | 1.15 | ornithine decarboxylase antizyme 2 |
| ILMN_1801119 | BCL2 | 596 | 0.000237549 | 0.876 | B-cell CLL/lymphoma 2 |
| ILMN_1716797 | CD302 | 9936 | 0.000243455 | 1.14 | CD302 molecule |
| ILMN_1734878 | CD79A | 973 | 0.000244235 | 0.731 | CD79a molecule, immunoglobulin-associated alpha |
| ILMN_1741406 | HOOK1 | 51361 | 0.000247298 | 0.886 | hook homolog 1 (Drosophila) |
| ILMN_1661366 | PGAM1 | 5223 | 0.000248881 | 1.123 | phosphoglycerate mutase 1 (brain) |
| ILMN_1669608 | PDE7A | 5150 | 0.000250397 | 0.877 | phosphodiesterase 7A |
| ILMN_1781560 | ST3GAL6 | 10402 | 0.00025508 | 1.138 | ST3 beta-galactoside alpha-2,3-sialyltransferase 6 |
| ILMN_1794165 | PGD | 5226 | 0.000257654 | 1.151 | phosphogluconate dehydrogenase |
| ILMN_1775672 | SOD2 | 6648 | 0.000259655 | 1.189 | superoxide dismutase 2, mitochondrial |
| ILMN_1751020 | PACSIN1 | 29993 | 0.000271021 | 0.881 | protein kinase C and casein kinase substrate in neurons 1 |
| ILMN_1669523 | FOS | 2353 | 0.000275723 | 1.202 | FBJ murine osteosarcoma viral oncogene homolog |
| ILMN_1653652 | PTPRC | 5788 | 0.000282898 | 1.135 | protein tyrosine phosphatase, receptor type, C |
| ILMN_1766613 | ZNF121 | 7675 | 0.000285806 | 0.899 | zinc finger protein 121 |
| ILMN_1795963 | OKL38 | 29948 | 0.000285911 | 1.155 | oxidative stress induced growth inhibitor 1 |
| ILMN_1740864 | TREML2 | 79865 | 0.000288918 | 1.164 | triggering receptor expressed on myeloid cells-like 2 |
| ILMN_1652787 | PIK3AP1 | 118788 | 0.000299051 | 1.123 | phosphoinositide-3-kinase adaptor protein 1 |
| ILMN_1739794 | CD3E | 916 | 0.000304133 | 0.88 | CD3e molecule, epsilon (CD3-TCR complex) |
| ILMN_1693630 | C16orf7 | 9605 | 0.000308826 | 1.155 | chromosome 16 open reading frame 7 |
| ILMN_1709237 | EPHX2 | 2053 | 0.000310605 | 0.84 | epoxide hydrolase 2, cytoplasmic |
| ILMN_1698224 | BCL11A | 53335 | 0.000310748 | 0.879 | B-cell CLL/lymphoma 11A (zinc finger protein) |
| ILMN_1791253 | FBXL13 | 222235 | 0.000319086 | 1.161 | F-box and leucine-rich repeat protein 13 |
| ILMN_1714592 | CDA | 978 | 0.000321033 | 1.257 | cytidine deaminase |
| ILMN_1781131 | UBN1 | 29855 | 0.000327957 | 1.173 | ubinuclein 1 |
| ILMN_1675406 | PPAPDC1B | 84513 | 0.000327961 | 0.871 | phosphatidic acid phosphatase type 2 domain containing 1B |
| ILMN_1801216 | S100P | 6286 | 0.00032979 | 1.401 | S100 calcium binding protein P |
| ILMN_1792689 | HIST1H2AC | 8334 | 0.000340877 | 1.144 | histone cluster 1, H2ac |
| ILMN_1696549 | BIRC3 | 330 | 0.000356635 | 0.848 | baculoviral IAP repeat-containing 3 |
| ILMN_1675117 | HSD17B11 | 51170 | 0.00035702 | 1.147 | hydroxysteroid (17-beta) dehydrogenase 11 |
| ILMN_1667319 | LPPR2 | 64748 | 0.0003606 | 1.174 | lipid phosphate phosphatase-related protein type 2 |
| ILMN_1683277 | KIAA0319L | 79932 | 0.000363149 | 1.124 | KIAA0319-like |
| ILMN_1798804 | SRPK1 | 6732 | 0.000369319 | 1.143 | SFRS protein kinase 1 |
| ILMN_1811077 | LHFPL2 | 10184 | 0.000376061 | 1.138 | lipoma HMGIC fusion partner-like 2 |
| ILMN_1770161 | BST1 | 683 | 0.00038223 | 1.114 | bone marrow stromal cell antigen 1 |
| ILMN_1717809 | RNF24 | 11237 | 0.000382433 | 1.236 | ring finger protein 24 |
| ILMN_1658121 | CFP | 5199 | 0.000390734 | 1.124 | complement factor properdin |
| ILMN_1707810 | RPS5 | 6193 | 0.000401819 | 0.854 | ribosomal protein S5 |
| ILMN_1698996 | SLC19A1 | 6573 | 0.000405413 | 1.144 | solute carrier family 19 (folate transporter), member 1 |
| ILMN_1701114 | GBP1 | 2633 | 0.000413057 | 1.247 | guanylate binding protein 1, interferon-inducible, 67kDa |
| ILMN_1776384 | ALDH16A1 | 126133 | 0.000413432 | 0.865 | aldehyde dehydrogenase 16 family, member A1 |
| ILMN_1683969 | FKBP1A | 2280 | 0.000413756 | 1.143 | FK506 binding protein 1A, 12kDa |
| ILMN_1704730 | CD93 | 22918 | 0.000418306 | 1.161 | CD93 molecule |
| ILMN_1800626 | SESN1 | 27244 | 0.000426184 | 0.889 | sestrin 1 |
