# Supplementary material for: Activated TLR Signaling in Atherosclerosis among Women with Lower Framingham Risk Score: The Multi-Ethnic Study of Atherosclerosis
Source: PLoS One. 2011 Jun 16;6(6):e21067. doi: 10.1371/journal.pone.0021067 (PMC3116882; doi:10.1371/journal.pone.0021067)
Supplement: Table S2 — Selected enriched pathways and functional categories. (DOC) [file pone.0021067.s005.doc]

**Table S2: Selected enriched pathways and functional categories**

**Ingenuity Pathway Analysis**

| Term | #Molecules | P-value | Genes |
| --- | --- | --- | --- |
| Immune response | 56 | 2.25E-16 | ADM,BCL2,C5AR1,CASP8,CCR7,CD27,CD93,CD3E,CD79A,CEACAM1,CEACAM3,CLEC7A,  CSF3R,F5,FCGR2A,FCGR3B,FKBP1A,FOS,FPR2,GBP2,GIMAP5,IL1B,IL1R2,IL1RN,IL6R,IL8RB,ITK,LAT2,MAPK14,NCF1,NCF4,NFIL3,OSM,PGLYRP1,PIK3AP1,PLCG1,PSG3,PTAFR,PTGS2,PTPRC,PTPRCAP,PXN,SEMA4D,SKAP1,STAT3,TLR1,TLR2,TLR4,TLR5,TLR6,TLR8,  TNFRSF10B,TNFSF14,TREM1,TREML2,ZAP70 |
| Inflammatory response | 37 | 4.33E-12 | C5AR1,CASP4,CCR7,CEACAM3,CSF2RA,CSF3R,CXCL16,EPHX2,FCGR2A,FKBP1A,FOS,  FPR2,GNAI3,IL1B,IL1RN,IL6R,IL8RA,IL8RB,IRAK3,MAPK14,MEFV,NCF1,OSM,PLAUR,  PTAFR,PTGS2,PTPRC,S100A12,STAT3,TLR1,TLR2,TLR4,TLR5,TLR6,TLR8,TNFSF14,TREM1 |
| Inflammation | 27 | 7.60E-11 | C5AR1,CASP4,CASP8,ENTPD1,FCGR2A,IL1B,IL1R2,IL1RN,IL6R,IRAK3,ITK,MAPK14,MME,  OSM,PTAFR,PTGS2,PTPRC,S100A12,SOD2,STAT3,TLR2,TLR4,TLR5,TLR8,TNFRSF10B,  TNFSF14,TREM1 |
| Proliferation of normal cells | 62 | 4.79E-12 | ADM,ARID3A,BCL2,CASP8,CCR7,CD6,CD27,CD3E,CD79A,CDC25B,CEACAM1,CSF2RA,  CSF2RB,CSF3R,CXCL16,DGKA,DUSP1,E2F3,FKBP1A,FOS,FPR2,GAB2,GBP1,GNAQ,ID3,IL1B,IL1RN,IL6R,IL8RB,IRS2,ITK,LEF1,LPAR2,LST1,MAGED1,MAPK14,NAMPT,NCF1,NOTCH1  ,OSM,PIK3AP1,PIK3IP1,PLAUR,PROK2,PTAFR,PTGS2,PTPRC,PTPRCAP,REM2,SEMA4D,  SKAP1,SOD2,STAT3,TCF4,TGFA,TIMP2,TLR2,TLR4,TLR5,TNFRSF10B,TNFSF14,ZAP70 |
| Mobilization of calcium | 27 | 3.36E-10 | C5AR1,CCR7,CD3E,CXCL16,FCGR2A,FCGR3B,FPR2,GAB2,GNAI3,GNAQ,IL8RA,IL8RB,ITK  ,ITPR3,LAT2,LILRA5,LPAR2,PIK3AP1,PILRA,PKN2,PLAUR,PROK2,PTAFR,PTPRC,TLR2,  TREM1,ZAP70 |
| Activation of leukocytes | 32 | 1.15E-09 | BCL2,C5AR1,CASP8,CD27,CD93,CD3E,CEACAM1,CEACAM3,CLEC7A,F5,FCGR2A,FKBP1A,  FOS,FPR2,GIMAP5,IL1B,IL1RN,IL8RB,ITK,LAT2,PTGS2,PTPRC,SEMA4D,STAT3,TLR2,TLR4,TLR5,TLR8,TNFRSF10B,TNFSF14,TREM1,TREML2 |
| Apoptosis | 75 | 1.45E-07 | ADM,B4GALT5,BCL2,BCL11B,BIN1,BIRC3,BLK,C5AR1,CASP4,CASP8,CD27,CD3E,CDC25B,CEACAM1,CSF2RA,CSF2RB,DUSP1,DUSP6,E2F3,ELL,FAIM3,FCER2,FKBP1A,FOS,FRAT1,  GAB2,GIMAP5,GNAQ,HK2,ID3,IL1B,IL1RN,IL6R,IL8RA,IL8RB,IRS2,ITK,ITPR3,LEF1,LPAR2,MAGED1,MAP4K1,MAPK14,MCL1,MEFV,MME,NAMPT,NCF1,NFIL3,NOTCH1,OSM,PADI4,  PGLYRP1,PIK3IP1,PKN2,PLAUR,PTAFR,PTGS2,PTPRC,PXN,REPS2,SOD2,SRPK1,ST8SIA4,  STAT3,TCF4,TGFA,TIMP2,TLR1,TLR2,TLR4,TLR6,TNFRSF10B,TNFSF14,TREM1 |

**DAVID Functional Analysis**

| Term | #Molecules | P-value | Genes |
| --- | --- | --- | --- |
| Immune response | 41 | 2.85E-12 | IL1R2,LST1,TLR1,TLR2,PGLYRP1,TNFSF14,TLR4,TLR5,LY9,TLR6,SKAP1,TLR8,CFP,CLEC4E,BCL2,LILRA5,ZAP70,IL1B,NFIL3,FCGR3B,CD27,PTPRC,C5AR1,BST1,NCF1,NCF4,IL1RN,IL6R,OSM,LAT2,CCR7,NOTCH1,CXCL16,FAIM3,CD79A,TREM1,CLEC7A,CD302,GBP2,PTAFR,  GBP1 |
| Response to wounding | 31 | 3.59E-09 | TLR1,TLR2,TLR4,TLR5,TLR6,TLR8,MMP25,CFP,FOS,DYSF,MEFV,BCL2,IL1B,ENTPD1,IL1RN,EPHX2,IL6R,STAT3,S100A12,PLAUR,SOD2,PROK2,CCR7,NOTCH1,F5,GNAQ,ADM,ID3,  CLEC7A,CD302,PTAFR |
| Defense response | 33 | 8.04E-09 | TLR1,PGLYRP1,TLR2,TLR4,TLR5,TLR6,PTPRCAP,TLR8,MMP25,CFP,FOS,MEFV,BCL2,CSF3R,IL1B,PTPRC,ITK,C5AR1,NCF1,IL1RN,EPHX2,PSG3,CLIC1,IL6R,STAT3,S100A12,PROK2,  CCR7,CXCL16,FAIM3,CLEC7A,CD302,PTAFR |
| leukocyte activation | 19 | 1.11E-07 | PTPRC,GIMAP5,CD3E,TLR1,TLR2,TNFSF14,FKBP1A,TLR4,TLR6,SKAP2,LAT2,CD93,BCL2,  BCL11B,BCL11A,ZAP70,TREML2,CLEC7A,CD79A |
| Inflammatory response | 21 | 4.67E-07 | TLR1,IL1RN,EPHX2,TLR2,TLR4,IL6R,TLR5,TLR6,TLR8,STAT3,MMP25,S100A12,CFP,FOS,  PROK2,CCR7,MEFV,IL1B,CLEC7A,CD302,PTAFR |
| I-kappaB kinase/NF-kappaB cascade | 10 | 8.68E-07 | TNFRSF10B,TLR1,TLR2,NLRP12,TNFSF14,TLR4,TLR5,TLR6,TLR8,CD27 |
| Toll-like receptor signaling pathway | 10 | 3.87E-04 | TLR1,TLR2,TLR4,TLR5,TLR6,TLR8,FOS,MAPK14,CASP8,IL1B |

**PANTHER Ontology Analysis**

| Term | #Molecules | P-value | Genes |
| --- | --- | --- | --- |
| Immune response | 33 | 2.67E-07 | CCR7,CLEC7A,FCGR3B,FCGR2A,CSF2RA,CSF2RB,CSF3R,EMR3,GBP1,GBP2,IL1B,IL1RN,  IL6R,IL8RA,IL8RB,TNFSF14,TNFRSF10B,TNFAIP2,BLR1,GAB2,CD27,VPREB3,STAT3,FAIM3,S100P,S100A12,LY9,CFP,ADARB1,LILRA5,RGR,NFIL3,SUSD3 |
| Toll receptor signaling | 8 | 9.13E-06 | IRAK3,TLR1,TLR4,TLR4,TLR6,TLR8,PTGS2,MAPK14 |
| Inflammation mediated by chemokine and cytokine signaling | 14 | 2.37E-04 | CCR7,BLR1,ITPR3,IL1B,PTAFR,PTGS2,STAT3,PLCG1,FPRL1,GNAQ,GNG10,C5AR1,IL8RA,  IL8RB |
| Apoptosis  Signaling | 8 | 9.50E-04 | CASP8,MCL1,TNFRSF10B,BCL2,HSPA6,MAP4K1,FOS,BIRC3 |
