# Supplementary material for: Activated TLR Signaling in Atherosclerosis among Women with Lower Framingham Risk Score: The Multi-Ethnic Study of Atherosclerosis
Source: PLoS One. 2011 Jun 16;6(6):e21067. doi: 10.1371/journal.pone.0021067 (PMC3116882; doi:10.1371/journal.pone.0021067)
Supplement: Table S3 — Correlation of gene expression between microarray and RT-PCR experiments. (DOC) [file pone.0021067.s006.doc]

| **Table S3: Correlation of gene expression between microarray and RT-PCR experiments** | | | |
| --- | --- | --- | --- |
|  |  |  |  |
| Gene | Pearson Correlation | P-value | P-value† |
| TNFSF14 | 0.5014 | 7.27E-09 | 8.79E-04 |
| TLR8 | 0.5023 | 6.77E-09 | 4.99E-05 |
| TLR4 | 0.6192 | 1.26E-13 | 6.24E-06 |
| CREB5 | 0.5949 | 1.21E-12 | 2.61E-03 |
| IL1B | 0.6491 | 1.78E-15 | 5.61E-04 |
| IL1RN | 0.5586 | 4.90E-11 | 2.40E-03 |
| EGLN1 | 0.5201 | 1.58E-09 | 5.96E-04 |
| TGFA | 0.4745 | 5.67E-08 | 1.12E-02 |

†T test for comparing groups with and without activated-immune

gene expression (AIGE) signature
