# Supplementary material for: Activated TLR Signaling in Atherosclerosis among Women with Lower Framingham Risk Score: The Multi-Ethnic Study of Atherosclerosis
Source: PLoS One. 2011 Jun 16;6(6):e21067. doi: 10.1371/journal.pone.0021067 (PMC3116882; doi:10.1371/journal.pone.0021067)
Supplement: Table S4 — Characteristics of 16 CHAS non-diabetic white men with low or high coronary artery calcium (CAC) score. (DOC) [file pone.0021067.s007.doc]

**Table S4: Characteristics of 16 CHAS non-diabetic white men with low or high**

**coronary artery calcium (CAC) score**.

| Variable | High CAC  (n=8) | Low CAC  (n=8) | P Value^*^ |
| --- | --- | --- | --- |
| Age, years | 71.0±4.5 | 70.9±4.9 | 0.95 |
| Body-Mass Index (Kg/m^2^) | 28.5±3.9 | 28.8±4.6 | 0.91 |
| Systolic BP (mm Hg) | 129.7±15.4 | 130.3±17.8 | 0.94 |
| Diastolic BP (mm Hg) | 71.7±10.6 | 79.2±10.4 | 0.18 |
| Total cholesterol (mg/dL) | 154.0±18.0 | 193.2±17.5 | <0.001 |
| LDL cholesterol (mg/dL) | 87.4±14.7 | 116.9±23.5 | <0.001 |
| HDL cholesterol (mg/dL) | 46.5±5.0 | 47.5±8.0 | 0.83 |
| Triglycerides (mg/dL) | 100.3±27.6 | 144.2±64.7 | 0.07 |
| hs-CRP (mg/L) | 1.27±0.76 | 1.21±0.40 | 0.84 |
| Current/former smoker (%) | 0/37.5 | 0/37.5 | 1 |
| Lipid-lowering medication use (%) | 75 | 25 | 0.05 |
| Anti-hypertension medication use (%) | 50 | 37.5 | 0.61 |

*p-value by t-test or chi-square test as appropriate.
